# Pancreatic islet cryopreservation by vitrification achieves high viability, function, recovery and clinical scalability for transplantation

**DOI:** 10.1038/s41591-022-01718-1

**Published:** 2022-03-14

**Authors:** Li Zhan, Joseph Sushil Rao, Nikhil Sethia, Michael Q. Slama, Zonghu Han, Diane Tobolt, Michael Etheridge, Quinn P. Peterson, Cari S. Dutcher, John C. Bischof, Erik B. Finger

**Affiliations:** 1 Department of Mechanical Engineering, University of Minnesota, Minneapolis, MN USA; 2 Department of Surgery, University of Minnesota, Minneapolis, MN USA; 3 Schulze Diabetes Institute, University of Minnesota, Minneapolis, MN USA; 4 Department of Chemical Engineering and Materials Science, University of Minnesota, Minneapolis, MN USA; 5 Department of Physiology and Biomedical Engineering, Mayo Clinic, Rochester, MN USA; 6 Center for Regenerative Medicine, Mayo Clinic, Rochester, MN USA; 7 Department of Biomedical Engineering, University of Minnesota, Minneapolis, MN USA

**Keywords:** Preclinical research, Stem-cell research, Diabetes

## Abstract

Pancreatic islet transplantation can cure diabetes but requires accessible, high-quality islets in sufficient quantities. Cryopreservation could solve islet supply chain challenges by enabling quality-controlled banking and pooling of donor islets. Unfortunately, cryopreservation has not succeeded in this objective, as it must simultaneously provide high recovery, viability, function and scalability. Here, we achieve this goal in mouse, porcine, human and human stem cell (SC)-derived beta cell (SC-beta) islets by comprehensive optimization of cryoprotectant agent (CPA) composition, CPA loading and unloading conditions and methods for vitrification and rewarming (VR). Post-VR islet viability, relative to control, was 90.5% for mouse, 92.1% for SC-beta, 87.2% for porcine and 87.4% for human islets, and it remained unchanged for at least 9 months of cryogenic storage. VR islets had normal macroscopic, microscopic, and ultrastructural morphology. Mitochondrial membrane potential and adenosine triphosphate (ATP) levels were slightly reduced, but all other measures of cellular respiration, including oxygen consumption rate (OCR) to produce ATP, were unchanged. VR islets had normal glucose-stimulated insulin secretion (GSIS) function in vitro and in vivo. Porcine and SC-beta islets made insulin in xenotransplant models, and mouse islets tested in a marginal mass syngeneic transplant model cured diabetes in 92% of recipients within 24–48 h after transplant. Excellent glycemic control was seen for 150 days. Finally, our approach processed 2,500 islets with >95% islets recovery at >89% post-thaw viability and can readily be scaled up for higher throughput. These results suggest that cryopreservation can now be used to supply needed islets for improved transplantation outcomes that cure diabetes.

## Abstract (web summary)

Optimization of vitrification approaches substantially improves pancreatic islet cryopreservation for banking and boosts transplantation outcomes in diabetes.

## Main

Despite 100 years of therapeutic development since the discovery of insulin, current diabetes therapies, such as continuous glucose monitors, insulin pumps and closed-loop systems, remain a treatment for the condition rather than a cure of the disease^1^. Although recent decades have seen substantial progress in the development of islet transplantation as a potential cure for diabetes^2^, one of the main limitations of this approach is that transplants from a single donor are often insufficient to achieve insulin independence in the recipient^3,4^. Frequently, two, three or more donor islet infusions totaling 700,000 to >1 M islet equivalents (IEQs) are required for a ‘typical’ 70-kg recipient^5,6^, adding risks associated with repeat surgical interventions and multiple rounds of strong immunosuppression induction.

One strategy for overcoming the donor supply problem is to pool islets from multiple donors, achieving high islet dosage with a single infusion^7,8^, increasing efficacy and reducing risk. Although several groups have shown the feasibility of culturing islets for extended periods (weeks to months)^9^, the majority have reported reduced islet recovery and loss of endocrine function over time^10,11^. Thus, large clinical trials often limit culture to 48–72 h before transplant^12^. This inability to culture or store high-quality islets for more than a few days after isolation, however, makes islet pooling logistically impractical. A second strategy is to develop an alternative islet source, such as SC-derived islets, which offer the exciting promise of an unlimited islet supply^13,14^ and decreased reliance on limited donor availability. SC-derived islets produce insulin in response to glucose, restore normoglycemia in some animal transplant models and have been tested in phase 1 and 2 trials in humans. However, heterogeneity in endocrine cell composition and variability in function^13^ lead to considerable batch-to-batch variability^15^, requiring extensive pretransplant validation of each lot, during which time SC-islets deteriorate in culture. The inability to store islets before use is a common challenge in both strategies. Longer-term storage is required to overcome the supply chain barriers limiting the availability of sufficient quantities of islets and to enable adequate quality assessment before transplant.

Cryopreservation, or the stabilization of biomaterials at an ultralow temperature (less than −150 °C), can achieve pooling, long-term banking, and off-the-shelf availability of viable cells and tissues^16,17^. Conventional cryopreservation techniques utilize slow cooling (i.e., <1 °C min^−1^) of biological substances to a dehydrated frozen state with the presence of extracellular ice. The addition of a low concentration (~2 M) of CPAs helps stabilize cells during cryopreservation and improves cellular viability. Despite extensive investigation (Supplementary Table 1 and the studies reviewed in Kojayanet al.^18^), challenges persist for islet cryopreservation due to ice-related injury (i.e., suboptimal viability), inability to achieve clinical scalability and use of non-clinically accepted components (i.e., fetal bovine serum) required to improve viability.

A promising alternative to existing conventional cryopreservation methods is ice-free vitrification; that is, rapid cooling of a biomaterial to a glass-like state^19,20^. To avoid ice formation, the cooling and subsequent warming rates need to exceed the critical cooling rate (CCR) and critical warming rate (CWR), respectively. Increasing CPA concentration (>4 M) can lower the required CCR and CWR to attainable levels but cause toxicity in cells and tissues, especially at higher temperatures (>4 °C). Thus, there is a critical balance point that avoids both injury from ice and from CPA toxicity while maintaining clinical scalability. Previous islet vitrification strategies were limited to small volumes with low islet quantities (<150 islets in microliter volumes of CPA solution; Supplementary Table 1) to ensure sufficient cooling and warming rates, and volumetric scale-up reduced the cooling and warming rates and led to ice formation, compromising viability. To our knowledge, no published technique has simultaneously achieved islet cryopreservation with high viability, function and recovery in a clinically scalable protocol (Supplementary Table 1).

**Fig. 1:**
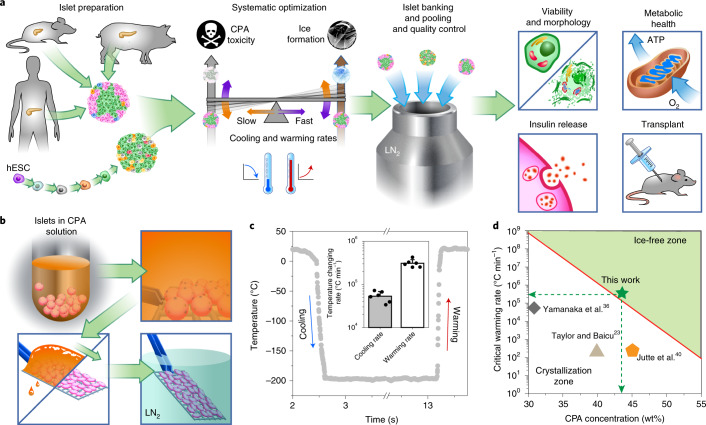
Overview of the study and the cooling/warming rates of cryomesh. **a**, Cryopreservation can be the cornerstone of an islet supply chain, allowing pooling, banking, and quality control before transplant. Model systems used to explore this include mouse, porcine and human islets and SC-beta islets. To achieve high recovery, viability, function and scalability simultaneously, systematic optimization of interrelated parameters, including CPA toxicity, ice formation and cooling and warming rates during VR cryopreservation, was performed in these islet systems. The achieved cooling and warming rates can adjust the balance between CPA toxicity and ice formation. Islet morphology, viability, metabolic health and in vitro and in vivo function were evaluated after VR cryopreservation. hESC, human embryonic stem cell. **b**, Schematic of cryomesh VR (not to scale). After CPA loading, islets in suspension were transferred to the cryomesh, and excessive CPA solution was removed before being plunged into liquid nitrogen (LN_2_). **c**, Representative measured temperature profile of cryomesh VR. Inset is the achieved cooling and warming rates (*n* = 6, data presented as mean ± s.d. with individual data points). **d**, Correlation of CWR and CPA concentration (equation (S2) in Supplementary Materials) indicates that ~44 wt% CPA is minimally required to avoid lethal ice using cryomesh VR and shows where other studies have failed to use a CPA with an adequate CWR to avoid ice under their thermal performance conditions.

To achieve this goal, we conducted systematic investigations on the CPA (formulation, concentration, loading and unloading) and vitrification method (enhancing cooling and rewarming rates) to develop a new islet cryopreservation method that simultaneously achieves high recovery, viability, functionality and scalability. We validated our methods on mouse, porcine, human and human SC-derived islets. Extensive in vitro assessments of viability, metabolic health and insulin secretion were performed. Finally, using syngeneic and xenogeneic transplant models, we demonstrated that after VR, islets remain viable in vivo, produce insulin, respond to glycemic challenge and cure diabetes. The overview of our technique is summarized in Fig. 1a.

## Results

### Preparation of pancreatic islets

For this study, we tested cryopreservation and postwarming function of mouse, porcine, human and SC-beta islets. SC-beta islets were clusters generated by differentiation of a human embryonic SC line (HUES8) in six stages using nongenetic programming while in 3D suspension culture^21^. Supplementary Fig. 1 shows an example of SC-beta cell-specific characterization during each stage of differentiation. Mouse islets and SC-beta islets were used for initial development, and the approach was then validated using human and porcine islets.

### Development of a scalable VR method

We first screened various VR approaches and assessed their performance in cooling, rewarming and scalability. We compared three commonly used droplet-based VR strategies: (1) copper dish cooling and convective warming, (2) copper dish cooling and laser nanowarming (gold nanorods were added to the droplet to generate heat upon laser irradiation) and (3) convective cooling and warming using a cryotop device (Supplementary Fig. 2; details in Methods). Briefly, 10–20 islets in a 2-µl droplet were vitrified and subsequently rewarmed. We assessed the cooling and warming rates by direct measurement or by estimation via modeling (Supplementary Table 2)^16^. Post-thaw viability was 56%, 62% and 55% using approaches 1, 2 and 3, respectively. Overall, each of these approaches suffered from suboptimal viability and potential challenges in scalability (details in Supplementary Results and Discussion).

To overcome these droplet-based limitations, we next tested a cryomesh system that can successfully cryopreserve *Drosophila melanogaster* embryos^22^. The cryomesh consists of a nylon mesh (38-µm pore size) attached to a plastic handle. After loading CPA in islets while in suspension, the islets were transferred to a cryomesh support, and excess CPA solution was removed by wicking; this is a critical step, as it reduces the bulk of the thermal mass in the system, leaving a thin layer of islets on the cryomesh surrounded by a minimal volume of CPA (Fig. 1b). By removing this excess fluid, the cooling and warming rates are increased by roughly an order of magnitude to 5.4 × 10^4^ and 30.9 × 10^4^ °C min^−1^, respectively, during convective cooling/warming (Fig. 1c). CPA removal also increases the density of islets within the system versus a droplet. For instance, a tightly packed monolayer of islets on a 2 cm × 2 cm nylon mesh (i.e., 1.7 × 10^4^ islets) is >95% of the volume on the cryomesh, whereas 20 islets in a 2-µl droplet occupy only 1.8% of the total volume. Supplementary Table 2 summarizes the performance of cryomesh, droplet and cryotop VR approaches, emphasizing both the improved cooling and warming rates and the ability to scale-up the cryomesh.

### CPA loading and unloading protocol

Having established the thermal performance of the cryomesh system, we sought a CPA formulation and loading/unloading protocol that would avoid ice formation while minimizing toxicity. Through a correlation between CPA concentration and CWR (equation (S2)), we estimated that the minimal CPA concentration in the interior of an islet should be 42–44 wt% to avoid devitrification (ice formation) during rewarming at 30.9 × 10^4^ °C min^−1^ using the cryomesh (Fig. 1d). CPA toxicity can occur from both chemical and osmotic injury. To avoid injury, we tested stepwise CPA addition to minimize osmotic damage (i.e., extreme volume shrinkage below 60% or volume swelling above 153%) during loading and unloading, also assessing chemical toxicity as a function of step durations and a variety of CPA formulations.

**Fig. 2:**
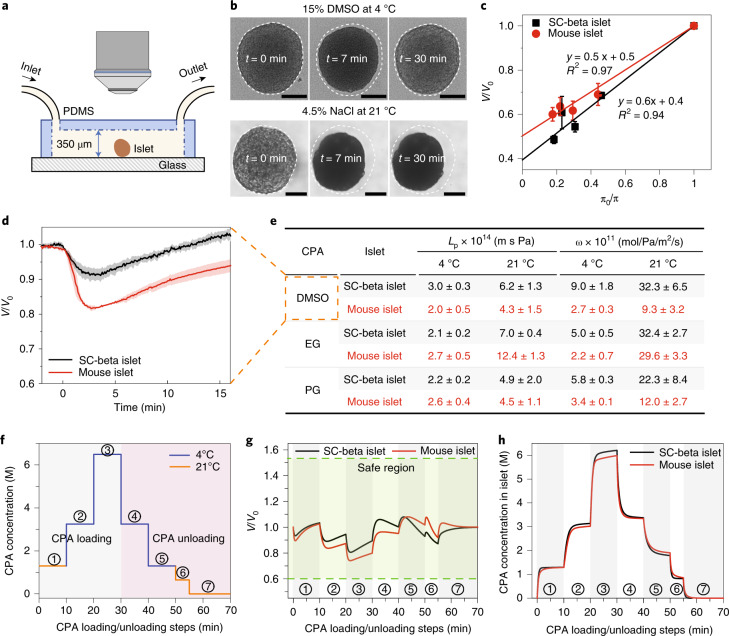
Islet biophysical property measurement and CPA loading/unloading protocol design. **a**, Schematic of the microfluidic device used to measure the biophysical properties of the islets (not to scale). The morphological changes of the islets were recorded via a microscope. PDMS, polydimethylsiloxane. **b**, Top: When subjected to 15 wt% DMSO at 4 °C, the islet first shrinks and then swells. Upon exposure to hypertonic CPA, water exits the cells, and the islet shrinks. CPA then diffuses across the cell membranes, followed by water re-entering the cell, leading to swelling back towards their initial state. Bottom: The islet remained shrunk in NaCl solution as the cells are impermeant to salt. Scale bars, 100 µm. **c**, Boyle–van’t Hoff plots of mouse and SC-beta islets display the normalized islet volume (*V/V*_*0*_) as a function of the osmolality ratio of isotonic and hypertonic NaCl solution (*π*_*0*_*/π*). The osmotic inactive volume (*V*_*b*_) that does not participate in the osmotic response can be estimated by extrapolating the linear fit to *π*_*0*_*/π* = 0. Further details can be found in Methods (*n* = 4 for SC-beta islets, *n* = 8 for mouse islets). **d**, Normalized volume of mouse and SC-beta islets versus time demonstrating the shrink–swell behavior when exposed to 15 wt% DMSO at 21 °C (*n* = 3 for mouse islets, *n* = 9 for SC-beta islets). **e**, Summary table of mouse and SC-beta islets water (*L*_p_) and CPA (ω) permeability at 4 °C and 21 °C (*n* = 3–9; the exact sample size can be found in Supplementary Fig. 3). The red color represents mouse islet in panels **c**,**d**,**g**,**h**, and is used in panel **e** to maintain consistency with the rest of the panels. **f**, Stepwise loading (steps 1–3) and unloading (steps 4–7) of 22 wt% EG + 22 wt% DMSO for islets. **g**, Modeled islet normalized volume change during CPA loading/unloading using the measured biophysical properties. The volume of both mouse and SC-beta islets remained in the safe region. **h**, Modeled CPA concentration in the mouse and SC-beta islets. For **c**–**e**, data are presented as mean ± s.d.

To develop an optimized loading and unloading CPA protocol, we first fabricated a microfluidic device to measure islet biophysical parameters, including osmotic inactive volume (*V*_*b*_), membrane water permeability (*L*_*p*_) and CPA permeability (*ω*). *V*_*b*_ was measured by recording the final equilibrium volume of islets in various concentrations of hypertonic NaCl solution (Fig. 2b, lower panel). The Boyle–van’t Hoff plots indicate that the osmotically inactive volume for mouse and SC-beta islets were 0.5 and 0.4, respectively (Fig. 2c). The membrane permeability parameters (*L*_*p*_, *ω*) were determined by recording the characteristic shrink–swell behavior of islets upon exposure to CPA solutions containing 15 wt% propylene glycol (PG), ethylene glycol (EG) and dimethyl sulfoxide (DMSO) at 4 °C and 21 °C (Supplementary Fig. 3). The ‘shrink’ occurs due to high water permeability upon initial CPA exposure, driving water efflux from the islets. This shrink is followed by a ‘swell’ as permeable CPA diffuses across the cell membrane and water slowly re-enters the islets (Fig. 2b, upper panel). We used the two-parameter model based on *L*_*p*_ and *P*_*s*_ (=*ωRT*, *R* is gas constant, *T* is absolute temperature) to fit the experimental shrink-swell curves (details in Methods). As shown in Fig. 2e, the water (*L*_*p*_) and CPA permeability (*ω*) have larger values at a higher temperature for both mouse and SC-beta islets. The different cellular compositions between mouse and SC-beta islets likely led to the difference in permeability values between the two islet types. Although the above describes the shrink–swell during loading, the inverse occurs during unloading, which is a swell followed by a shrink, as noted elsewhere^23^.

With these parameters (*V*_*b*_*, L*_*p*_ and *ω*), the intracellular CPA concentration, islet volume and chemical toxicity during a multistep loading and unloading were modeled (equations (2) and (3) in Methods, equation S1 in Supplementary Methods). After optimization, our CPA loading protocol used the following 10-min step intervals: 21 °C at 1.3 M, 4 °C at 3.2 M and 4 °C at 6.5 M (Fig. 2f; details in Supplementary Results and Discussion). The volume excursions of mouse and SC-beta islets remained within the osmotic tolerances (Fig. 2g). The model predicted CPA concentration to be 6.2 M inside mouse islets and 6 M for SC-beta islets (Fig. 2h).

### CPA formulation optimization

**Fig. 3:**
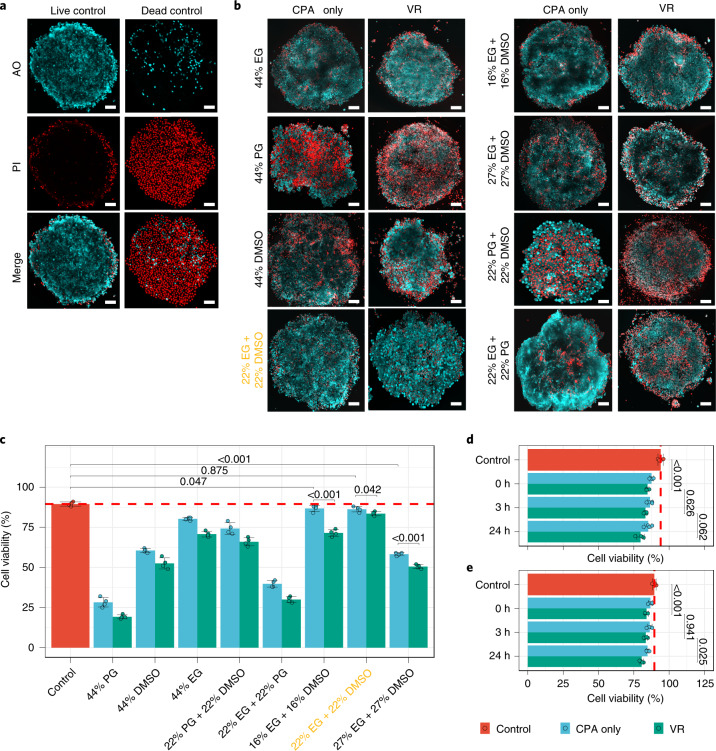
CPA formulation optimization for high post-VR viability. **a**, Confocal microscope images of live and dead controls of SC-beta islets stained with AO (cyan) and PI (red). **b**, Confocal microscope images (AO/PI merge) of CPA-treated (CPA loading and unloading only) and VR-treated (CPA loading, VR and CPA unloading) SC-beta islets. Various CPA formulations were examined. **c**, Cell viability of CPA-only-treated (cyan), VR-treated (green) and live control (red) SC-beta islets. Islets were dissociated into single cells, and viability was then measured. The yellow text in **b**,**c** is used to highlight the optimal condition. One-way analysis of variance (ANOVA) with Tukey post hoc test was used to compare groups, and *P* values from informative pairwise comparisons are shown (*n* = 4). **d**,**e**, For mouse (**d**) and SC-beta (**e**) islet cell viability after different culture time (0, 3 and 24 h) after CPA-only treatment and then after VR treatment. One-way ANOVA with Tukey post hoc test was used to compare groups, and informative pairwise comparisons are shown (*n* = 4). Scale bars, 100 µm. Data are shown as individual data points and mean ± s.d.

To optimize the CPA formulation, we tested three overall CPA concentrations (33%, 44% and 54%) consisting of PG, EG, DMSO or mixtures. We used qualitative and quantitative live/dead staining to measure SC-beta islet viability for each CPA after loading and unloading (Fig. 2f) and again after VR. Qualitative measurements were determined by confocal imaging of intact islets after staining with acridine orange (AO) and propidium iodide (PI) (Fig. 3a). For quantitative measurement, islets treated with CPA with or without VR were dissociated to single cells and stained with AO/PI, and the percentage of live (AO^+^/PI^−^) cells was counted (Fig. 3b). Viability measured after loading and unloading reflected the toxicity of CPA. Further decrease in viability after VR presumably reflected ice-related injury during cooling and rewarming.

After CPA loading and unloading, EG demonstrated the least islet toxicity of the three individual components, followed by DMSO and PG (Fig. 3c). When mixtures were used at the same total concentration (44%), a mixture of EG and DMSO provided the least toxicity (cell viability was 96.4% of control), outperforming EG or DMSO alone. Islet viability also remained high (93.3% of control) after VR and remained essentially unchanged over 24 h of post-VR culture (Fig. 3d,e). Decreasing the mixture concentration (to 34%) led to lower post-VR viability due to ice formation; increasing the concentration (to 54%) resulted in lower viability due to CPA toxicity (Fig. 3c). The best-identified CPA formulation (22% EG + 22% DMSO) was used throughout the study.

### Viability and morphology of VR islets

**Fig. 4:**
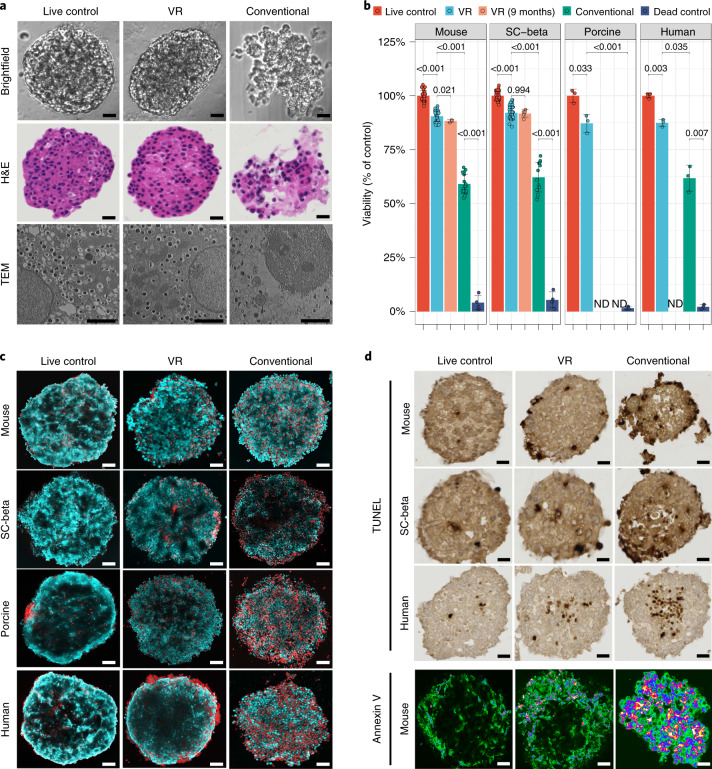
Viability and morphology of mouse, porcine, human and SC-beta islets following cryopreservation. **a**, Morphology of mouse islets from live control, VR (cryopreserved by VR) and conventional (cryopreserved by conventional slow freezing) was evaluated by brightfield microscopy, hematoxylin and eosin (H&E) histology, and TEM. For the conventional group, a mixture of intact and disrupted islets was observed. Examples of disrupted islet gross morphology due to ice formation are shown in the brightfield and H&E histology. **b**, Viability (percentage of live control) of mouse, SC-beta, porcine and human islets from treatment groups including live control, VR, VR 9 months (islets stored in LN_2_ for 9 months before rewarming), conventional (cryopreserved by conventional slow freezing) and dead control (treated by 75% ethanol). ND, not done. One-way ANOVA with Games–Howell post hoc test was used to compare groups, and *P* values from informative pairwise comparisons are shown (*n* = 3–34 per group; exact number in Supplementary Table 3). **c**, Confocal microscope images (AO/PI) of mouse, SC-beta, porcine and human islets from treatment groups, including live control, VR and conventional. **d**, TUNEL-stained images of mouse, SC-beta and human islets from treatment groups, including live control, VR and conventional. Bottom panel is Annexin V staining of mouse islets from the same treatment groups. Scale bars represent 2 µm for TEM, 50 µm for brightfield images, 70 µm for histology and TUNEL images and 100 µm for all fluorescence images. Data are shown as individual data points and mean ± s.d.

Using the optimized CPA formulation, loading and unloading conditions and cooling/rewarming cryomesh system, we examined islet morphology, viability, DNA fragmentation (TdT-mediated dUTP nick end labeling (TUNEL) stain) and Annexin V translocation following VR. We compared VR to healthy live control, dead control (ethanol-treated) and conventionally cryopreserved (i.e., slow cooling in 15% DMSO) islets (Fig. 4). Following VR, each of the four islet types (mouse, human, porcine and SC-beta) had an overall appearance and viability similar to fresh control islets and much better than conventionally cryopreserved islets (Fig. 4a). VR islets had smooth borders, rounded/oblong shape, and a normal histological appearance indistinguishable from fresh islets, whereas many conventionally cryopreserved islets demonstrated disruption of macroscopic architecture. The differences were even more evident on ultrastructural examination using transmission electron microscopy (TEM) (Fig. 4a, bottom). TEM imaging showed that the cell and nuclear membranes, mitochondria, secretory granules and other organelles appeared intact in VR islets. In contrast, conventionally cryopreserved islets had gross qualitative changes in cell appearance, reduced numbers of mitochondria and secretory granules and substantial blebbing of cellular and nuclear membranes.

We next used AO/PI staining to measure changes in viability associated with each islet cryopreservation technique. Qualitatively, VR islets appeared similar to live control islets, with only a slight increase in the number of red (necrotic or dead) cells (Fig. 4c and Supplementary Fig. 5) for each of the four islet types. Conventionally cryopreserved islets showed much more cell death. Following cryopreservation, a separate set of islets was dissociated into a single-cell suspension and tested for viability on a per-cell basis (Fig. 4b). Post-VR viability, relative to control, was 90.5% for mouse, 92.1% for SC-beta, 87.2% for porcine and 87.4% for human. The viability was unchanged over 9 months of cryopreservation (88.3% for mouse islets and 91.8% for SC-beta). Conventional cryopreservation resulted in lower viability (59.1–62.2%) than VR islets.

Although we primarily examined overall islet cell viability, to evaluate the viability of insulin expressing (beta) cells specifically, we costained intact or dissociated islets with fixable live/dead dyes and anti-insulin antibodies and then assessed beta cell viability by confocal microscopy (qualitative measures, Supplementary Fig. 6) and FACS (quantitative measures, Supplementary Fig. 7) and found that both insulin positive and negative cells appeared to have similar viabilities (details in Supplementary Results and Discussion).

In addition to the observed low degree of cell death (PI^+^ cells) with VR islets, we observed slightly more TUNEL^+^ and cell-surface Annexin V^+^ cells after VR in comparison to fresh control islets (Fig. 4d and Supplementary Fig. 8), suggesting both necrosis and apoptosis may contribute to the overall 8–12% decrease in viability with VR. Many more TUNEL^+^ and Annexin V^+^ cells were seen with conventionally cryopreserved islets. Compared with conventional cryopreservation, our VR technique resulted in substantial improvements in preserving the viability and morphology of the tested islet types.

### Metabolic health of VR islets

Because viability measured by membrane permeability dyes represents a lagging indicator of islet dysfunction following treatment, we sought to examine other measures of islet health that could better define the expected islet function following cryopreservation. Specifically, metabolic health, particularly islet OCR, is predictive of islet function in vivo^24,25^. We first examined ATP levels in islets immediately post-VR and found that, at that time point, ATP content was notably lower than in control islets (Supplementary Fig. 9). However, ATP levels and other metabolic measures (OCR and mitochondrial membrane potential) were largely recovered after 3 h of culture. We used that time point for all further assessments.

**Fig. 5:**
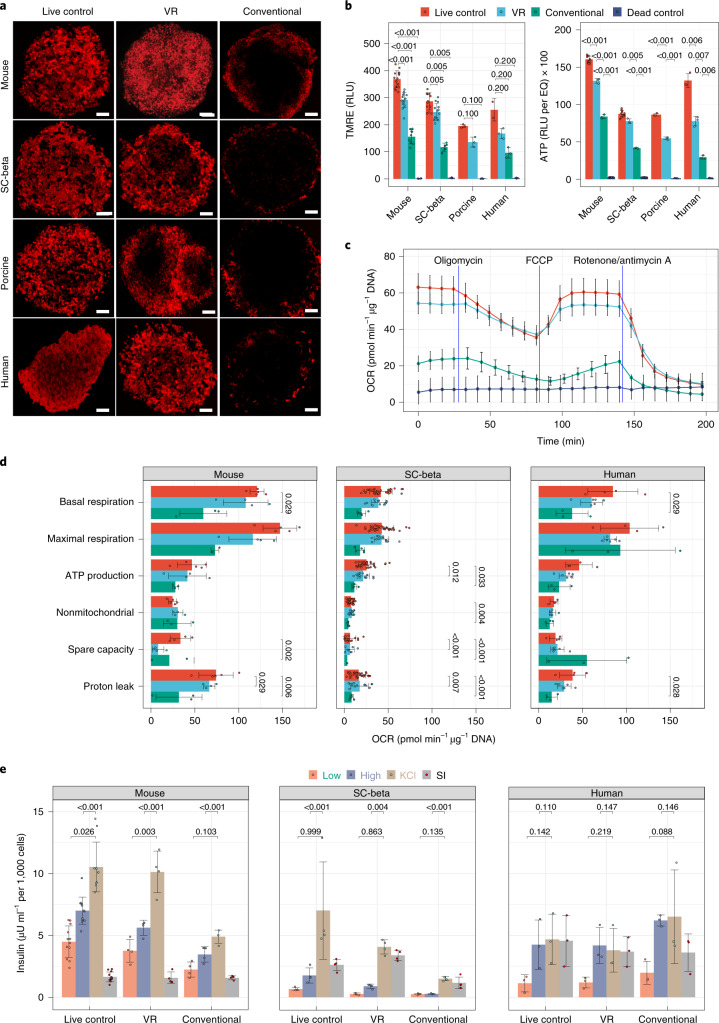
Metabolic health and in vitro function of islets following cryopreservation. **a**, Mitochondrial membrane potential (via TMRE staining) of mouse, SC-beta, porcine and human islets from treatment groups, including live control, VR and conventional. **b**, Left: Quantification of TMRE staining intensity. Comparisons shown between live control and treatment groups were performed by Kruskal–Wallis and pairwise Wilcoxon tests (*n* = 3–16 per group). Right: Measurement of ATP levels of four types of islets from live and dead control groups and cryopreservation groups (VR and conventional). One-way ANOVA with Games–Howell post hoc test was used to compare groups, and *P* values from informative pairwise comparisons are shown (*n* = 3–12/group). **c**, Example OCR curve showing the change in OCR during Mito Stress testing in SC-beta islets and comparing live control, VR, conventional cryopreservation and dead control islets (*n* = 5–8 per group at each time point). **d**, Compilation of the metabolic OCR parameters for each islet type and each treatment group. One-way ANOVA with Tukey post hoc test was used to compare groups, and significant (*P* < 0.05) pairwise differences are shown (*n* = 3–33 per group). **e**, In vitro GSIS assay for mouse, SC-beta and human islets from treatment groups, including live control, VR and conventional. One-way ANOVA with Tukey post hoc test was used to compare groups, and informative pairwise comparisons are shown (*n* = 3–12/group). In **b**, **d** and **e**, relevant statistical comparison *P* values are included within the plots. Scale bars, 100 µm. Data are shown as individual data points and mean ± s.d. For **b**–**e**, the exact number of replicates can be found in Supplementary Table 3. RLU, relative light units.

Mitochondrial membrane potential in each of the four islet types was measured by tetramethylrhodamine ethyl ester (TMRE) staining and qualitative and quantitative confocal microscopy. The overall signal intensity was somewhat lower for VR islets than live control (65.7–86.3% of control), but when adjusted for viable cell content, TMRE intensity was 75.1–93.7% of control (Fig. 5b, left). The difference in TMRE intensity may be due to slower recovery in the center of the islets where there was some central lucency. This appearance appeared to improve further with 24 h of culture (Supplementary Fig. 9). TMRE staining intensity for conventionally cryopreserved islets was 37–42% of control islets (Fig. 5b).

The number and appearance of mitochondria seen by TEM of VR islets were qualitatively similar to control islet cells (Fig. 4a, bottom). Similar to the TMRE findings, overall ATP levels in VR islets were slightly less than in the fresh control islets (Fig. 5b, right). These data suggest that cellular machinery to produce ATP remained intact, but ATP levels had not yet been fully restored to control values at the time point assayed.

Having shown intact mitochondria, we next looked at cellular respiration to assess whether VR islets were consuming oxygen to restore ATP. When compared to fresh control islets, VR islets showed a similar pattern of changes in OCR following stimulation with oligomycin (ATP synthase/complex V inhibitor), carbonyl cyanide 4-trifluorometheoxyphenylhydrazone (FCCP) (mitochondrial membrane uncoupling agent), and a mixture of rotenone (complex I inhibitor) and antimycin A (complex III inhibitor) (Fig. 5c). Conventionally cryopreserved islets showed a dampened OCR stress response. When comparing VR to fresh control in mouse, human and SC-beta islets, there were no differences in OCR for basal respiration, ATP production, proton leak, maximal respiration, spare respiratory capacity and nonmitochondrial respiration (Fig. 5d). These data suggest that VR islets maintain normal metabolic function as measured by cellular respiration.

### In vitro insulin secretion by VR islets

We performed GSIS testing to determine if islets were functional in vitro. Mouse, SC-beta and human islets secreted insulin in response to glucose challenge and, for mouse and SC-beta, further with complete depolarization using KCl (Fig. 5e). Porcine islets were not tested. Human islets had maximal insulin release with high-glucose challenge but no further release with KCl. The stimulation indices (ratio of insulin released with exposure of high-glucose concentration to that of low glucose) for VR islets of each islet type measured were not different from control islets. Conventionally cryopreserved islets also showed insulin release in response to glucose challenge, but to a lesser degree. These data confirm that VR islets are viable, metabolically active and functional in vitro.

### In vivo function of VR islets

**Fig. 6:**
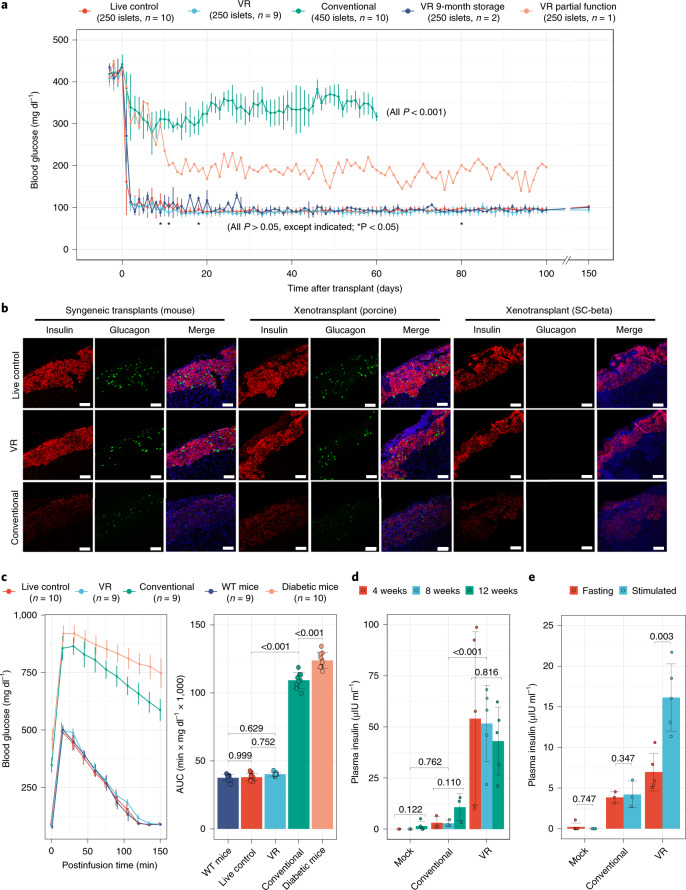
In vivo function of islets following cryopreservation. **a**, Blood glucose levels of streptozotocin-induced diabetic mice after syngeneic transplant of marginal mass mouse islets (250 islets per recipient) from treatment groups, including live control, VR, VR with 9-month cryopreserved storage (islets stored in LN_2_ for 9 months) and conventional cryopreservation (450 islets per recipient). All pairwise comparisons with *P* value <0.05 are shown (*P < 0.05), as determined by one-way ANOVA with Games–Howell post hoc test [*(n* = 10 (control and conventional cryopreservation), 9 (VR), 1 (VR partial function) and 3 (9-month storage and VR)). **b**, Insulin (red) and glucagon (green) staining in syngeneic (mouse) and xenogeneic (porcine and SC-beta) mouse transplant models. Treatment groups of islets include live control, VR and conventional. 4′,6-Diamidino-2-phenylindole staining (blue) is shown in the merged images. **c**, IPGTT of wild-type (WT) mice, diabetic mice and diabetic mice transplanted with live control, VR and conventional cryopreserved islets (left). Area under the curve (AUC) of IPGTT (right panel). Groups were compared by one-way ANOVA and Tukey post hoc test, and only informative pairwise comparisons are shown (*n* = 9–10 per group). **d**, Xenotransplant of SC-beta islets in NSG mice with nonfasting plasma human insulin levels at 4, 8 and 12 weeks after transplant. **e**, At 14 weeks, plasma insulin level of NSG mice after fasting and 30 min following stimulated insulin production by intraperitoneal glucose injection. For **d** and **e**, group comparison is by one-way ANOVA and Tukey post hoc test with informative comparisons shown (*n* = 3–5/treatment group). Scale bars, 200 µm. Data are shown as individual data points and mean ± s.d. For **c**–**e**, the exact number of replicates can be found in Supplementary Table 3.

As a final measure of post-VR islet function, we tested mouse, porcine and SC-beta islets in syngeneic and xenogeneic mouse transplant models (Fig. 6). Syngeneic mouse islets transplanted under the kidney capsule demonstrated function and restored normoglycemia within 48 h (Fig. 6a) and showed intense insulin immunofluorescence staining similar to control islets on posttransplant day 60, as did porcine and SC-beta xenotransplants in immunodeficient nonobese diabetic-*scid*-Il2rgc^−/−^ (NSG) recipients (Fig. 6b). Mouse and porcine islets also had intense glucagon staining, although potentially with a slight qualitative reduction in signal intensity compared with control islets. As expected, SC-beta transplants showed weak glucagon staining, as these had been differentiated to a beta cell lineage^21,26^. Conventionally cryopreserved islets had very low insulin or glucagon staining intensity.

Using a xenogeneic transplant model, we next tested the function of SC-beta islets in vivo. SC-beta islets were transplanted in nondiabetic NSG mice, and random serum samples were obtained to measure human insulin at 4, 8 and 12 weeks posttransplant (Fig. 6d). VR islets secreted human insulin throughout the 12 weeks. Conventionally cryopreserved SC-beta islets had detectable human insulin production, but these levels were not statistically different from mock transplant recipients. At 14 weeks, the recipient mice were tested for fasting and stimulated human insulin production by intraperitoneal glucose injection (Fig. 6e). Conventionally cryopreserved islet transplant recipients did have detectable human insulin, but there was no increase in levels following stimulation. VR islet recipients had higher fasting levels than mock or conventionally cryopreserved islets and demonstrated a 2.3-fold increase following stimulation, confirming SC-beta function in vivo.

Fresh control, VR and conventionally cryopreserved mouse islets were next tested in a marginal mass (250 islets per recipient) syngeneic islet transplant model using streptozotocin-induced diabetic recipients. Overall, VR islets rapidly restored normoglycemia in 92% (11/12) of recipients within 24–48 h (Fig. 6a). In bivariate Kaplan Meier assessment, time to normoglycemia was not different from live control islet transplant recipients (log-rank test, *P* = 0.063). One VR islet recipient demonstrated only partial function, potentially due to technical issues (i.e., islet leakage from kidney capsule). Blood sugar for that recipient fell below 200 mg dl^−1^ on posttransplant day 13 and then exhibited continued moderate hyperglycemia.

In contrast, conventionally cryopreserved islets failed to normalize blood glucose (BG) in all recipients, even with increased numbers of islets (450 islets per recipient). A subset of transplants underwent unilateral nephrectomy of the islet grafted kidney (Supplementary Fig. 10). Hyperglycemia developed rapidly in all mice, confirming that the transplanted islets, not the native recipient islets, controlled BG levels.

In the syngeneic transplants, glycemic control was extremely tight for VR islets. Random BG levels were not higher than those of control islet transplants throughout the posttransplant course (to 150 days posttransplant). To test the result of extended cryopreservation time, we vitrified islets, stored them in LN_2_ for 9 months and then rewarmed them. These long-term stored islet transplants also demonstrated rapid BG normalization and glucose stability throughout the follow-up period.

To demonstrate the level of glycemic control, we performed intraperitoneal glucose tolerance testing (IPGTT) on posttransplant day 50 (Fig. 6c). There were no differences in the glucose response curves (Fig. 6c, left) or the areas under the curves (Fig. 6c, right) for untreated wild-type mice, fresh control islet transplants and VR transplants. Recipients of conventionally cryopreserved islets had a glycemic response similar to diabetic control mice.

### Recovery and scalability

Islet recovery of our standard scale VR process (400–2,000 islets per test) was 96.8 ± 1.3% for mouse (*n* = 26), 96.6 ± 1.7% for SC-beta (*n* = 26), 91.3 ± 3.4% for porcine (*n* = 3) and 96.7 ± 1.5% for human (*n* = 6) islets (Supplementary Fig. 11). When we scaled up to medium throughput (2,500 islets per test), the recovery for mouse islets was 95.9 ± 1.2% (viability of 89.4%, *n* = 3), and SC-beta islet recovery was 98.2 ± 1.1% (*n* = 4). Finally, in our initial proof-of-concept test of higher throughput SC-beta islet VR (10,000 islets per test), recovery was 92.6% (*n* = 1).

Because the cryomesh system for VR is intrinsically unidimensional, scaling in the *x* and *y* dimensions is theoretically limited only by the container geometry. For these experiments, islets were vitrified on a 2 cm × 2 cm mesh at up to 4,250 islets per cm^2^. To achieve clinically meaningful throughput, units of 100,000 islets could thus be preserved on 24-cm^2^ cryomeshes.

## Discussion

Transplantation of a sufficient number of high-quality, viable and functional pancreatic islets into a diabetic patient can cure this increasingly common and progressively debilitating disease. A major limitation affecting the success of islet transplantation is the lack of an on-demand supply of sufficient numbers of high-quality native or SC-derived islets, a limitation that is exacerbated by the inability to store these cellular products before use. Here, we show, for the first time, an effective method for long-term islet preservation that achieves high viability, recovery, function and scalability simultaneously. Such a method for pancreatic islet banking via cryopreservation could revolutionize the supply chain for islet isolation, allocation, and storage before transplant, increasing the overall utilization of deceased donor pancreases and curing more patients of diabetes.

Prior studies explored both the conventional methods (slow cooling) and vitrification (rapid cooling) for the cryopreservation of islets, achieving varying degrees of success (Supplementary Table 1). For example, Rajotte et al.^27,28^ and other groups^29,30^ demonstrated the conventional cryopreservation of islets using a standard CPA (2 M DMSO) but achieved only low to moderate viability and function and at limited throughput (10–100 islets). Improvements in the protocol (that is, different CPA) resulted in better function^31,32^ or better throughput (≥10,000 islets)^33,34^, but not both simultaneously. Conventional cryopreservation was tested in limited clinical trials^35^ but was never fully adopted due partly to the use of fetal bovine serum, which comes with a risk of zoonotic infection. Meanwhile, several groups have tested vitrification (i.e., ice-free cryopreservation) as a favorable alternative to improve viability and function^23,36–39^. Initial studies demonstrated proof of principle, but with low islet throughput (50–300 islets) and partial function^23,40^. By changing the system’s geometry through the use of droplets^41^, hollow fibers^39^ or mesh supports^37,38^, other groups improved the cooling and heating rates and achieved modest improvements in function but at the cost of lower throughput (often just 25–100 islets at a time).

In addition to efficacy in cryopreserving freshly isolated mouse, porcine and human pancreatic islets, the VR approach was also effective for SC-derived human islets. SC-beta islets provide a promising source of human cells for beta cell replacement, demonstrating glucose-responsive insulin secretion in vitro and long-term glycemic control in diabetic mouse models^21,42^. Extensive efforts are underway to improve the SC-derived beta cell technology to achieve robust physiologic function; create efficient, low-cost differentiation methods; and improve the complement of endocrine cells (that is, produce with a mixture of alpha and beta cells)^43,44^. Nonetheless, a robust supply of high-quality SC-beta islets, such as enabled through cryopreservation, is still needed to realize off-the-shelf availability for future clinical adoption. Our cryopreservation method provides a potential solution, demonstrating high viability (92.1%) and in vitro and in vivo functionality of SC-beta islets, even after 9 months of storage in LN_2_. Long-term storage would allow immune manipulation of prospective recipients, enable comprehensive islet quality control before transplantation and increase cost efficiency by allowing large batches to be cryopreserved in functional single-patient units. These results suggest that our new cryopreservation protocol may be a powerful means of improving the SC-beta islet supply chain, thereby increasing therapeutic options for diabetic patients.

For both SC-beta islets and deceased donor islets, the scalability of the cryopreservation method is essential. Further scale-up of our approach for a clinical meaningful throughput (i.e., ≥100,000 islets), can be achieved using larger cryomesh sizes and alternative form factors such as mesh stacking (Supplementary Fig. 12). For a larger cryomesh size, when the mesh is plunged rapidly and uniformly, the cooling and rewarming fluxes and thermal mass (per unit area) will remain independent of mesh size and shape, under the conditions that cooling/rewarming baths are sized adequately (i.e., larger than the cryomesh) and the rewarming bath is agitated. In addition, the remaining CPA solution (entrained due to surface tension) between the islets and cryomesh temporarily ‘glues’ the islets to the cryomesh, and islets do not detach from the cryomesh during storage in LN_2_.

Our findings lay the groundwork for future clinical islet cryopreservation and suggest that high viability, functionality *(*in vitro and in vivo) and scalability can be achieved simultaneously during mouse, porcine, human and SC-beta islet cryopreservation. Further efforts are needed to address the limitations of our approach, including CPA optimization for human islets; further characterization of stress, adaptation and injury responses of islets; scale-up to a throughput of >300,000 islets per batch (typical yields for single-donor isolation are <300,000 IEQs^5^); and adaptation to clinical-grade processing (details in Supplementary Results and Discussion). We believe each of these limitations will be readily overcome in future development.

The implications of a successful method for islet cryopreservation before transplant are profound. Such technology has the following advantages: (1) improved efficacy through high-dose pooled donor islet transplants; (2) better opportunity for quality control and assessment; (3) better patient preparation by converting an unplanned operation into a planned event; (4) decreased risk by using a single islet infusion rather than repeated infusions; (5) improved opportunity for HLA matching of donor and recipient by selecting from a bank of available options rather than using the next donor in line; (6) facilitated tolerance induction protocols that require recipient preconditioning before transplants, such as tolerance achieved by apoptotic donor leukocytes infusion^45^ or mixed chimerism with islets and donor-derived hematopoietic SC transplant; and (7) improved organ utilization by promoting islet isolation and banking from all appropriate donors rather than only ‘optimal’ donors that have been historically selected to maximize yields in hopes of single-donor transplants.

## Methods

### Islet isolation methods

Institutional animal care and use committees from the University of Minnesota (protocol 1905-37028A) and the Mayo Clinic (protocol A00003973) approved the animal studies. Mice were housed with a 14-h on/10-h off light cycle, 68–74 °F ambient temperature and 30–70% humidity in specific pathogen-free (for NSG mice) or conventional (for C57BL/6 mice) facilities.

Mouse islets were isolated from C57BL/6 female retired breeders (age unspecified, Charles River Laboratories) by collagenase (CIzyme RI, VitaCyte) digestion and Histopaque (Millipore Sigma) density gradient enrichment^46^. Islets were then handpicked and cultured in a bioreactor (ABLE Biott reactor, BWV-S03A) in S3 media for 16 h before use. Human islets were purchased from a commercial vendor (Prodo Laboratories) and cultured in PIM(S) Complete media (Prodo Laboratories) for up to 7 days before use. Porcine islets were isolated from adult Landrace pigs^47^.

### SC-derived islet differentiation

HUES8 cells were cultured as spheroids in 500-ml spinner flasks (Corning, 3153)^21^. Suspension cultures were established by seeding 150 million cells (5 × 10^5^ cells ml^−1^) in mTeSR1 media (STEMCELL Technologies, 85850) with 10 µM Y27632 (R&D Systems, 1254) and maintained at 70 r.p.m. inside the humidified incubator at 37 °C, 5% CO_2_ and 100% humidity. Media was changed at 48 h to mTeSR1 without Y27632. Cells were passaged every 72 h by dispersing to single cells using Accutase (Millipore Sigma, A6964) with mechanical disruption and resuspended in fresh mTeSR1 with Y27632.

SC-beta differentiations were initiated after 3 days of SC spheroid formation and expansion in mTeSR1 media inside a spinner flask^21,26^. Clusters were allowed to settle by gravity, and the media was replaced with protocol-specific media including appropriate growth factors. Cell differentiation was directed sequentially to definitive endoderm, primitive gut tube, pancreatic progenitor 1, pancreatic progenitor 2, endocrine progenitors and finally into SC-beta^26^. Basal media types (S1, S2, S3 and BE5) were supplemented with inductive signals (details in the Supplementary Methods). The SC-beta islets were characterized by flow cytometry (details in Supplementary Methods).

### Measurement of islet biophysical parameters

The microfluidic devices used to measure islet biophysical parameters were fabricated using soft lithography procedures (details in Supplementary Methods). Handpicked islets were introduced into the device in retrograde fashion through the outlet hole. After initiating antegrade flow using islet media, CPA/salt solution of the desired concentration was loaded via a syringe pump. For CPAs, 15 wt% EG, PG and DMSO prepared in RPMI were used. For salt solutions, 1.8%, 2.7%, 3.6% and 4.5% sodium chloride prepared in deionized water were used. Once the islet settled on the channel floor, the CPA/salt solution flowrate was gradually increased while minimizing movement and rotation of the islet. The solution flow was stopped after at least five device volumes of solution had been pumped through the device. For experiments performed at 4 °C, the entire experiment was performed inside a cold room maintained at the same temperature. Islet cross-sectional area changes were recorded and analyzed using MATLAB to estimate islet spherical volume changes.

### Fitting of *L*_*p*_ and *ω*

A two-parameter model using *L*_*p*_ (hydraulic conductivity, also called water permeability) and *ω* (CPA permeability) was applied to fit the experimental shrink–swell data^48^. The assumptions are detailed in Supplementary Materials. The osmotic inactive cell volume, *V*_*b*_, was determined from the Boyle–van’t Hoff relationship^49^. The Boyle–van’t Hoff equation correlates the cell equilibrium volume with the osmolality of nonpermeating solution as below:

1 $$\frac{V}{{V_0}} = (1 - V_b)\frac{{\pi _0}}{\pi } + V_b,$$

where *V* is the cell equilibrium volume, *V*_*0*_ is the isotonic cell volume, *V*_*b*_ is the osmotically inactive volume fraction of the cell, *π*_*0*_ is the isotonic osmolality and *π* is the osmolality of the nonpermeating solution.

MATLAB was used to fit the *L*_*p*_ and *ω* of the mouse and SC-beta islets from the experimental shrink–swell curves. Modeling of islet volume change and intracellular CPA is based on the two-parameter ‘uncoupled’ model suggested by Kleinhans^48^, as follows:

2 $$\frac{{dV}}{{dt}} = - L_pART(C_S^e - C_S^i + C_C^e - C_C^i)$$

3 $$\frac{{dn_c}}{{dt}} = \omega RTA(C_C^e - C_C^i),$$

where *V* and *A* are the islet volume and surface area, respectively; *n*_*C*_ is the number of moles of CPA inside the islet; *R* is gas constant; *T* is the absolute temperature; *L*_*p*_ and *ω* are the hydraulic conductivity and membrane permeability to CPA, respectively; and *C* is the molality. The superscripts *i* and *e* denote intracellular and extracellular, respectively. The subscripts *C* and *S* denote permeating CPA and nonpermeating solutes, respectively.

### Islet cryopreservation

#### Conventional cryopreservation (slow cooling)

Islets were cryopreserved via a slightly modified slow freezing approach based on the previously established protocol by Rajotte et al.^50^. No fetal bovine serum or fetal calf serum was included in the CPA solution. At 21 °C, DMSO was added to the islet suspension to achieve a final concentration of 2 M in a cryovial. After a 25-min incubation with DMSO, the cryovial was placed in a −7.5 °C ethanol bath for 5 min. A metal rod was chilled in LN_2_ and used to seed ice by touching the suspension in the cryovial. After 15 min of latent heat of fusion release, the cryovial was cooled at 0.25 °C min^−1^ to −40 °C using a control rate freezer (Kryo 560, Planer Limited) and then plunged into LN_2_. Thawing was achieved using a 37 °C water bath. The thawed islets were placed in 0.75 M sucrose for 30 min at 0 °C to remove the intracellular CPA.

#### Cryomesh VR

The full-strength CPA used was 22 wt% EG + 22 wt% DMSO prepared in RPMI medium. To load the CPA, islets were first incubated in 20% CPA (i.e., 4.4 wt% EG + 4.4 wt% DMSO) for 10 min at 21 °C, followed by 50% CPA for 10 min at 4 °C and in 100% CPA for 10 min at 4 °C. The islet suspension was then transferred to the cryomesh placed on a wicking material (that is, paper towel). The CPA solution was wicked away through the nylon mesh and islets remained on the cryomesh. Clumping of islets was avoided. The cryomesh was quickly plunged into LN_2_ and stored in a LN_2_ tank. To thaw the islets, the cryomesh was plunged rapidly into the rewarming solution consisting of 11 wt% EG, 11 wt% DMSO and 5 wt% sucrose at 4 °C for CPA removal. After 10 min, the rewarming solution was diluted twofold using ice-cold 10 wt% sucrose solution, and the islets were incubated for another 10 min at 4 °C. The islets were then transferred to 21 °C, and the suspension was diluted twofold using 10 wt% sucrose solution. After 5 min, the islets were placed back in RPMI medium for 15 min as the last CPA removal step. At this time, all islets detached from the nylon mesh.

#### Cryotop VR

Using the same CPA loading procedure as in the cryomesh VR approach, 10–20 islets were included in a 2-µl droplet and placed on the cryotop. The cryotop was quickly plunged into LN_2_ for vitrification. The rewarming and CPA removal process was the same as that performed in the cryomesh VR.

#### Copper dish cooling and convective warming

A 2 µl droplet, including 10–20 islets, was dropped onto a copper dish floating in LN_2_ for vitrification. The vitrified droplet was convectively rewarmed in the unloading solution. The CPA removal steps were kept the same as in the cryomesh VR approach.

#### Copper dish cooling and laser nanowarming

Gold nanorods (nanoComposix) were added to the droplet to reach the concentration of 2.8 × 10^10^ parts ml^−1^. After vitrifying the islet and gold nanorod embedded droplet (2 µl) on the prechilled copper dish, a 1064-nm pulse laser was used to rewarm the droplet^17^. The pulse length was 7.5 ms, and laser voltage was 250 V. A high-speed camera (Nac Image Technology, Q1v) was used to record the laser warming process at 4,000 frames per second.

### TEM

The ultrastructures of islets were analyzed by TEM. Briefly, islets were fixed in 2.5% glutaraldehyde in 0.1 M sodium cacodylate buffer (pH 7.4) at 4 °C overnight and postfixed in 2% aqueous osmium tetroxide for 1 h, dehydrated in gradual ethanol (30% to 100%) and propylene oxide, embedded in Epon812 and cured for 48 h at 60 °C. Then, 65-nm ultrathin sections were collected onto 200-mesh copper grids and stained with uranyl acetate (15 min) and lead citrate (2 min) before examination by TEM. Images were captured with a FEI Tecnai G2 Spirit transmission electron microscope (Thermo Fisher Scientific).

### Viability assessment

Qualitative measurement of intact islet viability was performed using AO and PI. Intact islets were stained with 8 ng ml^−1^ AO and 20 ng ml^−1^ PI (Millipore Sigma) for 2 min at room temperature, coverslipped and imaged using an Olympus Fluoview 3000 inverted confocal microscope (Olympus) with 502/525-nm filters for AO and 493/636-nm filters for PI. The images were captured at 4,020 × 4,020-pixel resolution using a 20× magnification objective. Note that the islet diameters in all of the confocal images, here and throughout the study, are increased due to coverslip compression used to increase effective imaging depth.

Quantitative viability was measured on dissociated islet cells. After islet treatment, islets were incubated in a dynamic culture flask at 70 r.p.m., 37 °C, and 5% CO_2_ for 3 h in S3 media. The islets were dissociated into single-cell suspensions in TrypLE Express (Thermo Fisher Scientific, 12605010), quenched with S3 containing fetal bovine serum and stained with 8 ng ml^−1^ AO plus 20 ng ml^−1^ PI. After 15 s of incubation, 10 µl of the suspension was pipetted onto the Countess Cell Counting Chamber Slides (Thermo Fischer Scientific, C10228), and viability was quantified using a Countess II FL cell counter (Invitrogen by Thermo Fisher Scientific, AMQAF1000). The accuracy of the dissociated quantitative viability measurement technique was validated by comparing values to those obtained by image analysis of 3D reconstructions of confocal images of AO/PI-stained intact islets.

### Mitochondrial membrane potential measurements

Before performing the assay, islet clusters received were incubated in a dynamic culture flask at 70 r.p.m., 37 °C and 5% CO_2_. The islets were stained with 25 µl of 50 ng ml^−1^ reconstituted TMRE, perchlorate (Biotium, 70005) at room temperature for 1 min and 45 s on microscope slides, coverslipped (Chase Scientific, ZA0294) and imaged using an Olympus Fluoview 3000 confocal inverted microscope (excitation/emission filters: 594/574 nm). The images were captured at 4,020 × 4,020 resolution using a 20× magnification objective. The membrane potential of the islet was quantified by measuring fluorescence intensity using Olympus CellSens Dimension software (v1.17).

### ATP measurement

Islets were incubated in a dynamic culture flask at 70 r.p.m., 37 °C, and 5% CO_2_ before each assay. Standard-sized islets (~150 µm) were handpicked, and three IEQs per well were placed in each well of black 96-well plates (Greiner Bio-One, 89131-680) in 50 µl RPMI at 37 °C. Then, 50 µl prewarmed Promega CellTiter-Glo 3D cell viability reagent was added to each well. ATP standard (Roche) was used as positive control and calibrate the results. The plate was sealed, covered with aluminum foil and placed on an orbital shaker at room temperature for 5 min at 80 r.p.m. The plate was then incubated at room temperature for 25 min without shaking. A BioTek Synergy 2 Multi-Mode Microplate Reader was used to capture the luminescence, which was analyzed using the BioTek Gen5 software and reported as relative light units per IEQ.

### Cellular respiration/OCR measurement

Islets were incubated in a dynamic culture flask at 70 r.p.m., 37 °C and 5% CO_2_ for 3 h before the assay was performed. Cellular respiration was measured using the Agilent Seahorse XF Mito Stress Test and Agilent SeaHorse xFe24 Islet Capture FluxPak (Agilent, 103418-100) plates and grids. Islets were handpicked into wells containing 500 µl culture media in sufficient numbers to cover 50% of the inner circle of each sample well. The islet capture screen was carefully and securely fit onto to the plate. Islets were washed twice with SeaHorse media (SeaHorse XF DMEM) (Agilent, 103575-100) supplemented with 1 mM pyruvate, 2 mM glutamine and 5.6 mM glucose and equilibrated for 1 h at 37 °C. Assay reagents were loaded in a previously hydrated sensor cartridge. The assay plate was inserted into a calibrated Agilent SeaHorse xFe24 analyzer, and the Mito Stress test was performed according to the manufacturer’s protocol with the following optimized reagent concentrations: mouse islets (5 µM oligomycin, 1 µM FCCP and 10 µM each rotenone and antimycin A), SC-beta islets (10 µM oligomycin A, 2 µM FCCP and 10 µM each rotenone and antimycin A), human islets (50 µM oligomycin A, 10 µM FCCP and 10 µM each rotenone and antimycin A) and porcine islets (50 µM oligomycin A, 2.5 µM FCCP and 10 µM each rotenone and antimycin A). OCR values were obtained over 200 min. If the sampling rate differed between plates, the time course was scaled over the standard observation period.

For normalization between wells, the islets in each well were lysed in 1 M ammonium hydroxide + 0.2% Triton X-100. The DNA content was measured by PicoGreen assay (Molecular Probes) using standardized calibration controls for quantification on a BioTek Synergy 2 Multi-Mode Microplate Reader (BioTek). Individual cellular respiration parameters, including OCR for basal respiration, ATP production, proton leak, maximal respiration, spare respiratory capacity and nonmitochondrial respiration, were calculated and normalized to the DNA content obtained for individual wells.

### TUNEL staining

Islet clusters received were incubated in a dynamic culture flask at 70 r.p.m., 37 °C and 5% CO_2_ before the assay was performed. According to the manufacturer’s protocol, apoptotic cells were stained using ApopTag Peroxidase in situ Apoptosis Detection Kit (Millipore Sigma, S7100). Following staining and washing, the slide was mounted by dehydrating with xylene, and a coverslip was applied using mounting media. The number of apoptotic cells in each islet was counted under a light microscope.

### Annexin staining

Islet clusters received were incubated in a dynamic culture flask at 70 r.p.m., 37 °C and 5% CO_2_ before the assay was performed. Then, 5× Annexin V binding buffer (Biotium, 99902) was diluted in deionized water to obtain 1× binding buffer. Islets were washed twice using 1× binding buffer. The staining solution was prepared by diluting Annexin V conjugate in 1× binding buffer to a final concentration of 2.5 µg ml^−1^ and incubated with the islets at room temperature for 15–30 min, protected from light. The islets were then washed with 1× binding buffer three times and imaged within 30 min using the Olympus Fluoview 3000 confocal inverted microscope with an excitation/emission of 490/515 nm under a 20× objective. The Ca^2+^ phospholipid-binding protein with a high affinity for phosphatidylserine was quantified by gating the false-color mapping fluorescence using Olympus CellSens Dimension software (v1.17) and statistically analyzed.

### GSIS assay

GSIS assays were conducted to assess in vitro function of islets^26^. Briefly, islets were washed twice in low-glucose (3.3 mM glucose) Krebs Ringer buffer (KRB) (128 mM NaCl, 5 mM KCl, 2.7 mM CaCl_2_, 1.2 mM MgSO_4_, 1 mM Na_2_HPO_4_, 1.2 mM KH_2_PO_4_, 5 mM NaHCO_3_, 10 mM HEPES and 0.1% FAF-BSA in deionized water). The islets were then loaded into 24-well transwell inserts (Millicell, cell culture insert, PIXP01250) and fasted in low-glucose KRB for 1 h at 37 °C. Islets were washed once in low-glucose KRB and then incubated in low-glucose KRB for 1 h at 37 °C. The volume of the KRB with low glucose, high glucose and KCl was 1 ml per well. After incubation, the supernatant was collected and stored at −20 °C until analysis. The islets were then transferred to high-glucose KRB (16.7 mM) for 1 h at 37 °C, and the supernatant was collected and stored. The islets were then transferred to low-glucose KRB with 30 mM KCl to observe depolarization conditions and incubated in this buffer for 1 h, and the supernatant was collected. Finally, the islets were dispersed via incubation with TrypLE and counted using a Countess automated cell counter (Thermo Fisher Scientific). Collected supernatants were analyzed by enzyme-linked immunosorbent assay for human insulin concentrations (ALPCO, 80-INSHUU-E01.1) and normalized for cell number. Mouse islet insulin was measured by HTRF (homogeneous time resolved fluorescence) assay (Cisbio PerkinElmer, 62IN1PEG).

### Syngeneic mouse islet transplantation and IPGTT

C57BL/6 mice (6- to 8-week-old males, Charles River Laboratories) were rendered diabetic by single-dose (220 mg kg^−1^) streptozotocin (Millipore Sigma, S0130) intraperitoneal injection. Blood glucose levels were measured after 4–7 days, and diabetes was confirmed by two successive daily measurements of >400 mg dl^−1^. Marginal mass islet transplants of 250 islets per recipient^51^ were performed under the recipient mouse’s left kidney capsule. For all treatment groups, islets were randomly selected for transplantation, including islets of all sizes, both morphologically disrupted and intact islets. BG measurements were made daily. Transplant success was measured on the first day of two successive daily measurements of BG < 200 mg dl^−1^. Graft failure was defined as the first day of two consecutive measurements >250 mg dl^−1^.

On posttransplant day 50, IPGTT was performed by fasting mice for 16 h (overnight) and then injecting 2.5 g kg^−1^ glucose intraperitoneally and measuring BG every 15 min for 150 min.

To confirm that the transplanted islets were controlling BG levels and not a restoration of native beta cell function, left nephrectomy (including islet graft) was performed for a subset of transplants at posttransplant day 60, and return of hyperglycemia was verified. Day-60 explants were fixed and stained for insulin and glucagon.

### Xenogeneic islet transplantation and IPGTT

SC-beta and porcine islets were transplanted under the kidney capsule of nondiabetic 6- to 12-week-old male (NSG) mice (Jackson Laboratory)^21^. Briefly, islet clusters containing a total of 5 × 10^6^ cells were injected under the kidney capsule of male NSG mice. Control mice underwent a mock surgery in which saline was injected into the kidney capsule. Posttransplant facial bleeds were performed at weeks 4, 8 and 12 to measure human insulin levels. At 14 weeks posttransplant, modified IPGTT was performed. Mice were fasted for 16 h, and blood was collected before and 30 min after intraperitoneal injection of 2.5 g kg^−1^ glucose bolus. Serum was separated from blood using microvettes (Sarstedt, 20.1292.100), and human insulin was quantified using the human ultrasensitive insulin enzyme-linked immunosorbent assay (ALPCO, 80-INSHUU-E01.1). Following IPGTT, the graft-containing kidneys were explanted and stained for insulin and glucagon.

For porcine islet xenotransplants, 350 islets were transplanted under the kidney capsule of NSG mice (male or female, age 6–12 weeks). At 60 days posttransplant, the graft-containing kidneys were removed, fixed and stained for insulin and glucagon.

### Insulin and glucagon immunofluorescence labeling

Recovered kidneys with transplanted islets under the kidney capsule were fixed in 70% alcoholic formalin (BBC Biochemical, 0460) and embedded in paraffin blocks. Then, 5-µm sections were cut using a microtome. The sections were deparaffinized using xylene and decreasing 100%, 90%, 80% and 70% EtOH concentrations. Slides were incubated with blocking buffer (5% bovine serum albumin (Millipore Sigma, B6917) in DPBS with Ca^2+^ and Mg^2+^ (Thermo Fisher Scientific, 14040141)) for 20 min and then stained with species-specific reagents as follows. For beta cell-specific viability experiments, intact mouse islets were stained with fixable live dead dye (Biotium, Live-or-Dye NucFix) before fixation, permeabilization and staining with anti-insulin antibodies (see below).

#### Syngeneic mouse islet transplants

Kidney sections were incubated at room temperature with FLEX polyclonal guinea pig anti-insulin antibody (Agilent, IR002) for 1 h at room temperature. The sections were gently washed five times using the blocking buffer and then incubated with recombinant rabbit anti-glucagon antibody (Abcam, ab92517) in blocking buffer (1:570) for 1 h. The sections were then washed five times gently with blocking buffer and incubated with goat anti-guinea pig immunoglobulin G (IgG) (H+L) secondary antibody (Alexa Fluor 488) (Abcam, ab150185; 1:250) and goat anti-rabbit IgG H&L (Alexa Fluor 647) (Abcam, ab150079) in blocking buffer (1:250) for 1 h at room temperature. After gently washing the sections with 4 °C DPBS with Ca^2+^ and Mg^2+^, the sections were labeled with 4′,6-diamidino-2-phenylindole (Millipore Sigma, F6057), coverslipped (Chase Scientific, ZA0294) and placed at 4 °C for 2 h before imaging.

#### Porcine islet and SC-beta xenotransplants

Kidney sections were incubated at room temperature with mouse monoclonal anti-insulin antibody (K36aC10) (Abcam, ab6995) in blocking buffer (1:50 dilution) and recombinant rabbit anti-glucagon antibody (Abcam, ab92517) in blocking buffer (1:570) for 1 h at room temperature. The sections were then rinsed five times gently with blocking buffer and incubated with goat anti-mouse IgG H&L (Alexa Fluor 647) (Abcam, ab150115) in blocking buffer (1:250) and goat anti-rabbit IgG H&L (Alexa Fluor 488) (Abcam, ab150077) in blocking buffer (1:300) for 1 h in room temperature. After gently washing the sections with 4 °C DPBS with Ca^2+^ and Mg^2+^, the sections were labeled with 4′,6-diamidino-2-phenylindole (Millipore Sigma, F6057), coverslipped (Chase Scientific, ZA0294) and held at 4 °C for 2 h before imaging on an Olympus Fluoview 3000 inverted confocal microscope.

### Statistics and reproducibility

Statistical analysis was performed in R version 4.0.3 (R Foundation for Statistical Computing). We included the exact number of independent experiments (*n*) and technical replicates for each relevant figure in Supplementary Table 3. Normality was tested with the Shapiro–Wilk test for continuous variables or graphically using qq plots and distribution histograms. Homogeneity of variance was assessed using Levene’s test. For normal or near-normal group comparisons, ANOVA testing with pairwise post hoc Tukey HSD test was used to determine statistical differences. For groups with unequal variance, the Games–Howell test was used. Nonnormal variables were tested using the nonparametric Kruskal–Wallis test for overall significance and the pairwise Wilcox (Mann–Whitney *U*) test for individual group comparison. P values were adjusted for multiple comparisons. Time to event analysis was performed using the Kaplan–Meier method with the log-rank test of significance. Data are presented as mean ± s.d. unless specifically detailed otherwise. Full statistical treatment for each figure is presented in the Supplementary Data 2. Statistical testing was two sided, and a *P* value of <0.05 was considered significant.

### Reporting Summary

Further information on research design is available in the Nature Research Reporting Summary linked to this article.

## Online content

Any methods, additional references, Nature Research reporting summaries, source data, extended data, supplementary information, acknowledgements, peer review information; details of author contributions and competing interests; and statements of data and code availability are available at 10.1038/s41591-022-01718-1.

## Supplementary information

Supplementary Information Supplementary Results and Discussion, Methods, Figures 1–12, Tables 1–3 and References.

Reporting Summary

Supplementary Video 1 High-speed video (4,000 frames per second) of laser nanowarming (7.5-ms laser pulse) of a 2-μl droplet encapsulated with islets.

Supplementary Data 2 Summary of statistical treatment for each figure, including *P* values, test statistics and confidence intervals.

## Data Availability

All raw data have been archived for public access (10.13020/yrva-zr31)^52^.

## Code availability

R version 4.0.3 was used for statistical analysis. MATLAB 2018b was used for calculating volume changes of islets from the experimentally recorded shrink–swell videos. MATLAB 2019a was used for the shrink–swell curve fitting and chemical toxicity cost–function evaluation. The custom codes are available upon request from the corresponding authors.
