# Supplementary material for: Pancreatic islet cryopreservation by vitrification achieves high viability, function, recovery and clinical scalability for transplantation
Source: Nat Med. 2022 Mar 14;28(4):798–808. doi: 10.1038/s41591-022-01718-1 (PMC9018423; doi:10.1038/s41591-022-01718-1)
Supplement: Supplementary file 1 — Supplementary Results and Discussion, Methods, Figures 1–12, Tables 1–3 and References. [file 41591_2022_1718_MOESM1_ESM.pdf]

nature portfolio

https://doi.org/10.1038/s41591-022-01718-1

Supplementary information

Pancreatic islet cryopreservation by vitrification achieves high viability, function, recovery and clinical scalability for transplantation

In the format provided by the authors and unedited

Supplementary Materials for

Pancreatic islet cryopreservation by vitrification achieves high viability, function, recovery, and clinical scalability for transplantation

Li Zhan[1#], Joseph Sushil Rao[2, 3#], Nikhil Sethia[4], Michael Q. Slama[5], Zonghu Han[1], Diane Tobolt[2], Michael Etheridge[1], Quinn P. Peterson[5,6], Cari S. Dutcher[1,4], John C. Bischof[1,7†], Erik B. Finger[2†]*

[1]Department of Mechanical Engineering, University of Minnesota; Minneapolis, MN, 55455, USA.

[2]Department of Surgery, University of Minnesota; Minneapolis, MN, 55455, USA.

[3]Schultze Diabetes Institute, University of Minnesota; Minneapolis, MN, 55455, USA.

[4]Department of Chemical Engineering and Materials Science, University of Minnesota; Minneapolis, MN, 55455, USA.

[5]Department of Physiology and Biomedical Engineering, Mayo Clinic; Rochester, MN, 55902, USA.

[6]Center for Regenerative Medicine, Mayo Clinic; Rochester, MN, 55902, USA.

[7]Department of Biomedical Engineering, University of Minnesota; Minneapolis, MN, 55455, USA.

# These authors contributed equally to this work.

† These authors jointly supervised this work.

*Corresponding author. Email: efinger@umn.edu.

This file includes:

Supplementary results and discussion

Supplementary methods

Figures S1-S12

Tables S1-S3

References

Supplementary Results and Discussion

Performances of droplet-based VR approaches

For approaches 1-3, the cooling rates were similar: 1.1×10^4, 1.1×10^4, and 1.4×10^4 °C/min. The warming rates were 1.2×10^4, 31.3×10^4 (by modeling), and 2.3×10^4 °C/min, respectively (Table S2). Approach 1 (convective rewarming) had the slowest rewarming rate but more potential for scaling. In approach 2 (laser rewarming), the vitrified droplet was rapidly melted within the 7.5 milliseconds laser pulse (Supplementary Movie 1). Laser nanowarming, however, is not readily scalable due to decreasing viability in larger droplets because of a droplet lensing effect[1], and because of procedural complexity and high cost, which would hinder clinical deployment[2]. Approach 3 (cryotop cooling and convective rewarming) was intermediate in both rewarming and scalability.

CPA loading/unloading and formulation optimization

To avoid osmotic injury, the volume change of the islets was controlled to within 60% (i.e., shrink) and 153% (i.e., swell) of the initial volume[3]. To minimize chemical toxicity, we used a lower temperature (4 °C) for CPA concentrations higher than 2 M. In addition, we applied a chemical toxicity cost function to model the effect of step duration (Equation S1)[4,5]. Specifically, during the 3-step loading process, the duration of each step can be adjusted to reach the same final CPA concentration in islets. We found that the protocol with a medium step duration (10 min) had the lowest theoretical chemical toxicity cost function value compared with shorter or longer step durations (Fig. S4).

Our optimization of CPA formulation suggested that a cocktail of EG and DMSO has minimal toxicity, potentially due to toxicity neutralization effect[6,7]. Our CPA loading and unloading design showed minimal osmotic and accumulative chemical damages using the measured islet biophysical parameters and model-derived CPA loading protocol. For example, the viability of SC-beta islets remained at 96.4% after loading and unloading of 22 wt% EG + 22 wt % DMSO. Notably, we showed high viabilities post VR for porcine (87.2%) and human islets (87.4%), even though the protocol was optimized for mouse and SC-beta islets.

Viability of insulin expressing cells after VR

We primarily measured overall islet cell viability and did not routinely assess viability specifically in insulin expressing (i.e., beta) cells. However, we did find similar viability between beta and non-beta cells post VR in mouse and SC-beta islets upon testing (Figs. S6 and S7). In qualitative confocal imaging of mouse islets, the insulin expressing cells (Fig. S6, red cytoplasm) showed slight increase in cell death (blue nuclei) compared with fresh control islets, but much less death than conventionally cryopreserved samples. In quantitative assessment of SC-beta islet cell viability by FACS (Fig. S7), the beta cell specific viability (insulin$^+$) after VR was 80.0 ± 1.2%, which was not different than the viability of insulin$^-$ (non-beta) cells (77.3 ± 3.5%, $p = 0.272$, $n = 3$). Note that the overall viability (normalized to fresh control for insulin$^+$ and insulin$^-$ populations) was reduced during the more extensive cell dissociation used for these experiments.

In addition, we can indirectly assume through the largely equivalent function of fresh control and VR islets in GSIS, transplant, and IPGTT assays that the beta cell viability must be at least on par with the overall cellular viability (typically >90%). In addition, while we did not specifically quantify the total insulin content of VR islets as compared to fresh control, we can indirectly compare these groups by looking at the following measures: sum of the total insulin release in GSIS assays (sum of basal insulin and that produced

in response to high glucose challenge and KCl depolarization) (Fig. 5e), anti-insulin confocal images of explanted islet transplant grafts (Fig. 6b), glycemic control and IPGTT of mouse islet transplants (Fig. 6a and c), and in vivo glucose stimulated insulin release (Fig 6e). Each of these measures indicated that the total effective insulin content per islet was not significantly changed by VR.

Comparison with other islets cryopreservation methods

We implemented a holistic, integrative optimization of physical and biological parameters to improve VR outcomes and address the supply chain challenges for high quality and quantity of pancreatic islets. The CPA concentration and formulation, loading and unloading procedure, and cooling and warming rates must be carefully balanced. Assuming that we can exceed the CCR of the CPA used, the rate-limiting step to avoid damaging ice formation during VR is rewarming, which needs to exceed the CWR of that specific CPA at the concentration (wt %) used. This limit is illustrated in Fig. 3c when the cryomesh (warming rate ~ 3×10^5 °C/min) was unsuccessful in rewarming vitrified SC-beta islets loaded with 32 wt% CPA (CWR ~ 10^7 °C/min). Here the rewarming rate was lower than the CWR and the viability was low (79.9%). In contrast, by rewarming islets loaded with 44 wt% CPA, the CWR was decreased to a value that could be achieved with cryomesh rewarming (~ 10^5 °C/min), and viability increased to 92.1%. Even at 44 wt% CPA, cryotops that can warm at only ~2×10^4 °C/min were unsuccessful, with the viability dropping to 55% for mouse islets (Table S2).

With conventional cryopreservation method, many groups have documented suboptimal success rate or delayed time to normoglycemia (several days to weeks) using transplant models (Table S1). For improved outcome, the work of Cattral et. al. employed 10% fetal bovine serum (FBS) in the CPA solution and hand-picked islets with 100-150 µm diameter for transplant, showing normoglycemia at day 3-4 posttransplant in most mice[8]. In contrast, for comparison with our VR approach, we excluded FBS in the CPA solution (to reduce risk of zoonotic infection and in anticipation of cGMP-compliant translation) and randomly selected islets of all sizes and morphology (i.e., both disrupted and intact) to prepare conventionally cryopreserved islets for transplantation. We found that mice transplanted with conventionally cryopreserved islets resulted in lower blood glucose levels than STZ-induced control mice, but failed to achieve normoglycemia (i.e., partial endocrine function, Fig. 6a). It is possible that through further optimization we could have improved on the success of other strategies in our hands, but our approach led to a combination of viability, function, and clinical scalability not achieved using other methods.

None of the previous attempts in the literature have shown both high viability and functionality (in vivo and in vitro) when scaling up to more than >2,000 islets per cryopreservation session (Table S1). Lakey et al., for instance, found decreased functionality (i.e., in GSIS assays) for conventional cryopreservation when a larger container (i.e., 500 ml cryobag) was used[9]. Indeed, non-uniform cooling and warming rates within the larger container (i.e., center vs. edge) could lead to cryo-damage from thermal stress-induced fracture and lethal ice formation. For vitrification, Langer et al. reported low viability (i.e., 17%) when 10,000 islets were rewarmed at 200 °C/min using CPA consisting of 20.5% DMSO + 10% PG + 15.5% acetamide[10]. Not surprisingly, the achieved warming rate of 200 °C/min is substantially lower than the estimated CWR (> 10^4 °C/min) of the 46 % CPA cocktails they used, leading to poor viability. We demonstrated for the first time that both high viability and functionality were achieved when 2,500 islets were vitrified and rewarmed, and have shown in principle that this can scale to units of at least 10,000 islets per mesh.

Adaptation to clinical-grade processing

For potential clinical translation, one potential implementation would be to have donor pancreases transported to regional cGMP isolation facilities (centers of excellence) for isolation, cryopreservation, and storage. Cryopreserved islets (potentially from multiple donors) would then be allocated to specific recipients, transported to the cGMP facility serving the recipient transplant center, rewarmed by that cGMP facility, and transplanted. The equipment needed for the vitrification is relatively modest, and apart from a LN_2 sterilization system, is already present in most cGMP facilities. Similarly, the rewarming requires even less application-specific equipment and, with sufficient training, could be performed locally. Other options such as processing by national or commercial facilities could provide alternative workflows.

One important consideration of this approach is that the islets come into direct contact with LN_2 and sterility concerns will need further study. UV radiation has been shown to successfully kill microorganisms that can otherwise survive in liquid nitrogen[11] and, over the past few decades, cryopreservation of human oocytes and embryos using open-systems (i.e., Cyrotop) has been used extensively in the in vitro fertilization (IVF) industry, with no published reports of cross-contamination via direct contact with LN_2 during cooling[12,13]. Contamination issues could be further reduced by minimizing direct exposure with the liquid nitrogen and storing cryopreserved materials in hermetically sealed containers. Additionally, further development will be needed to avoid or reduce risks of exposure to bacterial endotoxin in the LN_2 system. Such strategies will likely include some combination of surface decontamination, use of disposable containers or container inserts, production of endotoxin free LN_2 gas, and multiple washes of islets to reduce endotoxin to FDA-accepted limits.

Supplementary Methods

Stem cell-derived islets differentiation

The following basal media types (S1, S2, S3, and BE5) were supplemented with inductive signals for stem cell-derived islets differentiation.

S1 media was comprised of 1L MCDB 131 (Life Technologies, 10372019) supplemented with 0.44 g glucose, 2.46 g sodium bicarbonate, 20 g fatty-acid-free bovine serum albumin (FAF-BSA, Proliant Biologicals, 68700), 20 µL ITS-X (Life Technologies, 51500-056), 10 mL Glutagro (Corning, 25-015-Cl), 44 mg ascorbic acid, and 10 mL penicillin/streptomycin (P/S) solution (30-001-Cl). S2 media: 1L MCDB 131 supplemented with 0.44 g glucose, 1.23 g sodium bicarbonate, 20 g FAF-BSA, 20 µL ITS-X, 10 mL Glutagro, 44 mg ascorbic acid, and 10 mL P/S. S3 media: 1L MCDB 131 supplemented with 0.44 g glucose, 1.23 g sodium bicarbonate, 20 g FAF-BSA, 5 mL ITS-X, 10 mL Glutagro, 44 mg ascorbic acid, and 10 mL P/S. BE5 media: 1L MCDB 131 supplemented with 3.6 g glucose, 1.754 g sodium bicarbonate, 20 g FAF-BSA, 5 mL ITS-X, 10 mL Glutagro, 44 mg ascorbic acid, 10 mL P/S, and 4000 units heparin (MilliporeSigma, H3149).

Directed differentiation of pluripotent stem cells to SC-beta cells was performed by changing media within the spinner flask and supplementation with small molecules and growth factors specific to the differentiation stage. Media changes are as follows: Day 1: S1 media + 100 ng/mL Activin A + 3µM CHIR99021; Day 2: S1 media + 100 ng/mL Activin A; Day 4: S2 media + 50 ng/mL KGF; Day 6: S3 media + 50 ng/mL KGF + 250 nM Sant-1 + 500 nM PDBu + 200 nM LDN 193189 + 2 µM RA + 10 µM Y27632; Day 7: S3 media + 50 ng/mL KGF + 250 nM Sant-1 + 500 nM PDBu + 2 µM RA + 10 µM Y27632; Days 8, 10, 12: S3 media + 50 ng/mL KGF + 250 nM Sant-1 + 100 nM RA + 10 µM Y27632 + 5 ng/mL Activin A; Days 13 + 15: BE5 media + 250 nM Sant-1 + 20 ng/mL betacellulin + 1 µM XXI + 10 µM ALK5i + 1 µM T3 + 100 nM RA; Days 17 + 19: 20 ng/mL betacellulin + 1 µM XXI + 10 µM ALK5i + 1 µM T3 + 25 nM RA; Days 20-26: S3 media only.

KGF (cat # 100-19) was purchased from Peprotech. All other factors were purchased from R&D Systems with the following catalog numbers: Activin A (338-AC), CHIR (4423), SANT-1 (1974), PDBu (4153), RA, Retinoic Acid (0695), LDN, LDN193189 (6053), Y27632 (1254), Betacellulin (261-CE), ALK5i (3742), T3, L-3,3',5-Triiodothyronine (5552), and XXI, γ-secretase inhibitor XXI (6476).

Flow cytometry for SC-beta characterization

Spheroids were dispersed into single cells by incubation in TrypLE Express at 37°C for 10 minutes and mechanically disrupted. Cells were quenched with S3 media, washed with PBS, fixed in 4% PFA for 60 min, and stored at 4°C in PBS until further use. For beta-cell specific viability experiments, dissociated clusters were stained with live/dead dye (LIVE/DEAD Fixable Near-IR, ThermoFisher, Waltham, MA), washed, and fixed prior to fixation. Before staining, cells were filtered through a 40 µm cell strainer, permeabilized in blocking buffer (1x PBS, 0.1% Triton X-100, 5% donkey serum) at room temperature for 40 minutes, and washed three times in PBST (0.1% Triton X-100). Cells were then incubated with primary antibodies in the blocking buffer for 1 hour at room temperature, washed 3 times with PBST, and incubated with secondary antibodies in blocking buffer for 1 hour at room temp. After labeling, cells were washed 3 times, resuspended in PBST at a concentration of 1 x 10^6 cells/mL, and analyzed using an Attune flow cytometer (ThermoFisher). Data were acquired and analyzed using NXT v. 3.1 (ThermoFisher) and FlowJo v.10 (FlowJo, Ashland, OR) software.

The following primary antibodies were used for flow cytometry: goat anti-PDX1 (R&D Systems, AF2419), mouse anti-NKX6.1 (DSHB, F55A12), rabbit anti-chromogranin (Abcam, ab15160), rat anti-c-peptide (DSHB, GN-ID4), and mouse anti-glucagon (Sigma, G2654). Secondary antibodies used for flow cytometry were donkey anti-goat Alexa Fluor 488 (Invitrogen, A-11055, 1:1,000), donkey anti-rabbit Alexa Fluor 488 (Invitrogen, A-21206, 1:1,000), donkey anti-rat Alexa Fluor 488 (Invitrogen, A-21208, 1:1,000), and donkey-anti-mouse-Alexa Fluor 647 (Invitrogen, A-31571, 1:1,000).

Fabrication of microfluidic devices for islet biophysical parameter measurement

A 350 µm thick SUEX epoxy film sheet (DJ MicroLaminates, Sudbury, MA) was bonded to the surface of a silicon wafer using hot-roll lamination. The sheet was then photopatterned with the channel geometry, generating a mold for creating subsequent devices. After exposing the wafer to silane vapors in a desiccator for 20 minutes, a 10:1 by weight mixture of polydimethylsiloxane (PDMS) prepolymer and curing agent (SYLGARD 184 Silicone Elastomer Kit, Dow Corning Corporation, US) was poured over the mold and incubated overnight at 70 °C for cross-linking. PDMS devices were cut out from the wafer and holes for tubing connections were punched using a 1.5 mm OD biopsy punch (Integra LifeSciences, Princeton, NJ). The PDMS device and glass slide were sealed after activating the bonding surfaces using oxygen plasma (Harrick Plasma, Ithaca, NY) at 18W for 1 minute and then stored at 70 °C for 2 hours. To render the PDMS channel walls hydrophilic, the device was treated with oxygen plasma for 15 minutes. The devices were soaked in water until usage to maintain channel hydrophilicity. The devices were mounted on the top of an inverted microscope for use, and the channels were flushed with the culture medium.

Assumptions for modeling of water and CPA transport in islets

The water and CPA transport phenomena in the islets are described by the Kedem-Katchalsky (K-K) equation[14]. The assumptions include that the cell membrane has constant lumped properties[15] and that irreversible thermodynamics can be applied to study the nonelectrolyte solute (CPA) and solvent (water) transports[14]. The hydraulic conductivity is a measure of the mechanical filtration capacity of the membrane or the velocity of water moving through the membrane per unit pressure difference, and the CPA permeability is a measure of the permeable solute transport across the membrane[16]. Both parameters are temperature dependent, following the Arrhenius relationship[17]. Islets are composed of thousands of heterogenous cells in a roughly spherical shape. For simplicity, we modelled an equivalent single osmotic unit with the same surface area and volume to represent the actual islet, assuming the CPA diffusion inside the islet and the heterogeneous permeability characteristics of cells don't affect the measured volume changes[16]. Considering the assumptions mentioned above, our modeling provides an estimation, rather than specific measurement of actual islet volume change during CPA loading and unloading process.

Chemical toxicity cost function

To minimize the chemical toxicity, we adapted the toxicity cost function developed by Benson et al. to evaluate the effect of step duration of CPA loading and unloading. Briefly, the accumulative temperature and concentration-dependent chemical toxicity can be quantitatively described by the J_{tox}, as shown below[18].

$$
\begin{cases}
k = \beta \cdot C_{CPA}^{\alpha} \\
\dfrac{dN}{dt} = -k \cdot N \\
J_{tox} = \int_0^{t_f} k\ dt = \int_0^{t_f} \beta \cdot C_{CPA}^{\alpha}\ dt \\
\dfrac{N}{N_0} = \exp(-J_{tox})
\end{cases}
\tag{S1}
$$

where k is toxicity rate, α and β are two constants that depend on the type of CPA, C_{CPA} is the intracellular CPA concentration, N is the viability after exposure, N_0 is the initial viability, t_f is the duration of CPA exposure, and J_{tox} is the toxicity cost function.

Using the SC-beta islets as the model system, we tested short, medium, and long CPA loading step durations that achieve the same final CPA concentration (6.2M) inside the islets (Fig. S4). For short CPA loading duration, 3 min in 20% CPA at 21 °C, 11 min in 50% CPA at 4 °C, and 11 min in 100% CPA at 4 °C; for medium CPA loading duration, 10 min in 20% CPA at 21 °C, 10 min in 50% CPA at 4 °C, and 10 min in 100% CPA at 4 °C; for long CPA loading duration, 15 min in 20% CPA at 21 °C, 35 min in 50% CPA at 4 °C, and 5 min in 100% CPA at 4 °C. We used $\alpha = 1.6$ and $\beta = 0.005$ for DMSO to calculate J_{tox}[18].

Measurement of cooling and warming rates

A type T thermocouple (COCO-002, OMEGA, Norwalk, CT) and an oscilloscope (DS1M12, USB Instrument) were used to measure the cooling and warming rates of various VR approaches. During the cryomesh VR, the thermocouple was attached to the cryomesh, and islets were added such that the thermocouple junction was in contact with the islets. The temperature was recorded during the convective cooling and warming processes. During the cryotop VR, the thermocouple was placed in the 2 μL droplet to measure the temperature changing rates. During the copper dish cooling and convective warming, the thermocouple junction was placed at ~1 mm from the surface of the copper dish[2]. The 2 μL droplet was dropped onto the thermocouple, and the temperature profile at the top of the droplet was recorded. Cooling and warming rates were calculated based on the temperature profile ranging from -140 °C to -20 °C. Notably, the measured sample will be in a glassy phase at -140 °C. The estimated critical warming rate (CWR) can be described as a function of CPA concentrations as below[19]:

$$CWR = 1.644 \times 10^{17} e^{-63.94C} \tag{S2}$$

Where C is the CPA concentration (w/w). For instance, $C = 0.44$ for 44% CPA concentration.

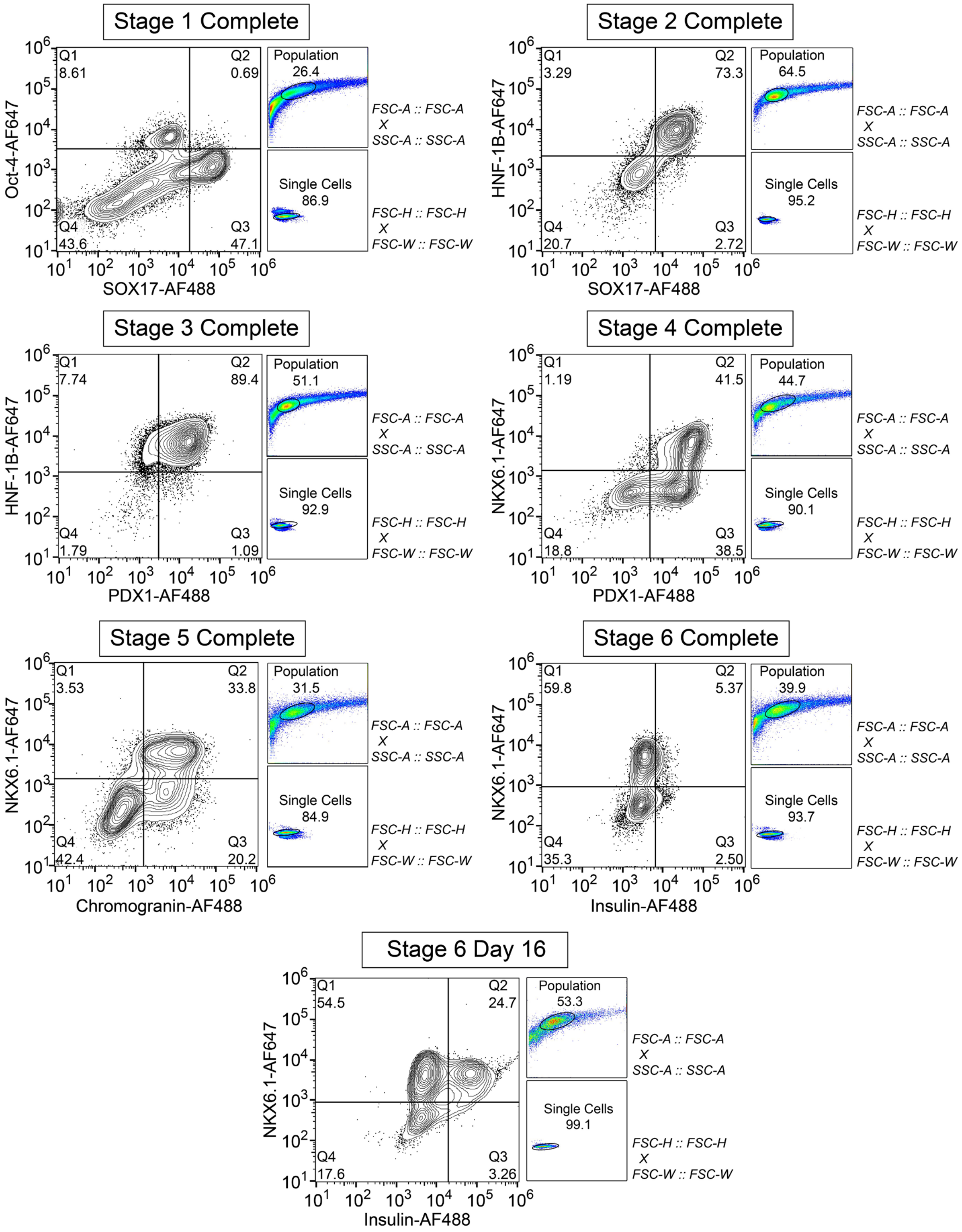

Fig. S1. In vitro differentiation of human stem cell (SC)-derived islets. SC-derived beta cell clusters (SC-beta) were differentiated from a human embryonic cell line (HUES8) in vitro. Differentiation is performed in 6 stages by programmed application of small molecules and growth factors.[21] Flow cytometry plots show the relevant changes in cell-specific markers (Oct-4, SOX17, HNF-1B, PDX1, NKX6.1, Chromogranin, and Insulin) through the stages of differentiation along with the gating strategy. The final SC-beta islet clusters contain ~25% Insulin+/NKX6.1+ beta cells.

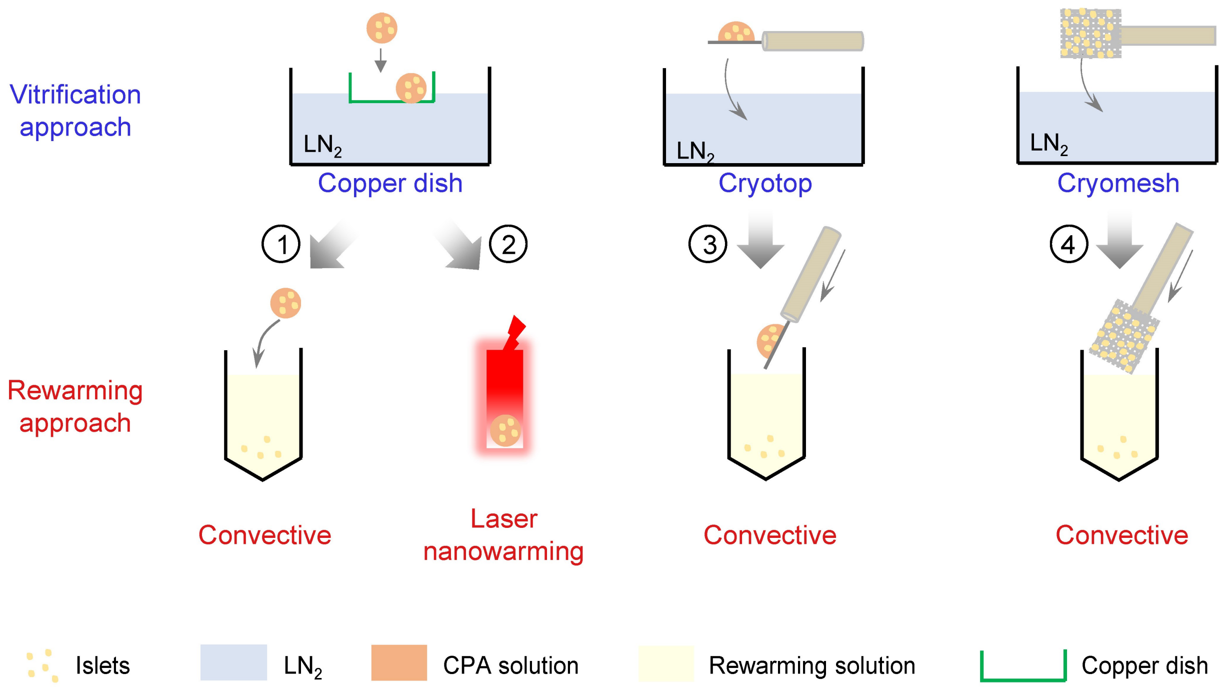

Fig. S2. **Schematics of 4 different Vitrification-Rewarming (VR) approaches tested for islet cryopreservation.** In approach 1, islets in 2 µl CPA droplets are dropped onto a copper dish floating on liquid nitrogen (LN_2) to achieve vitrification. The vitrified droplet is convectively rewarmed in the rewarming solution. In approach 2, gold nanorods are added to the CPA droplet. The islets are vitrified using the copper dish and rewarmed by laser irradiation. In approach 3, islets in a 2 µl CPA droplet are loaded onto the cryotop. The cryotop is plunged into LN_2 for vitrification and into the rewarming solution for warming. In approach 4, islets are transferred to the cryomesh, and the CPA solution is wicked away. The cryomesh is plunged into LN_2 for vitrification and into the rewarming solution for warming.

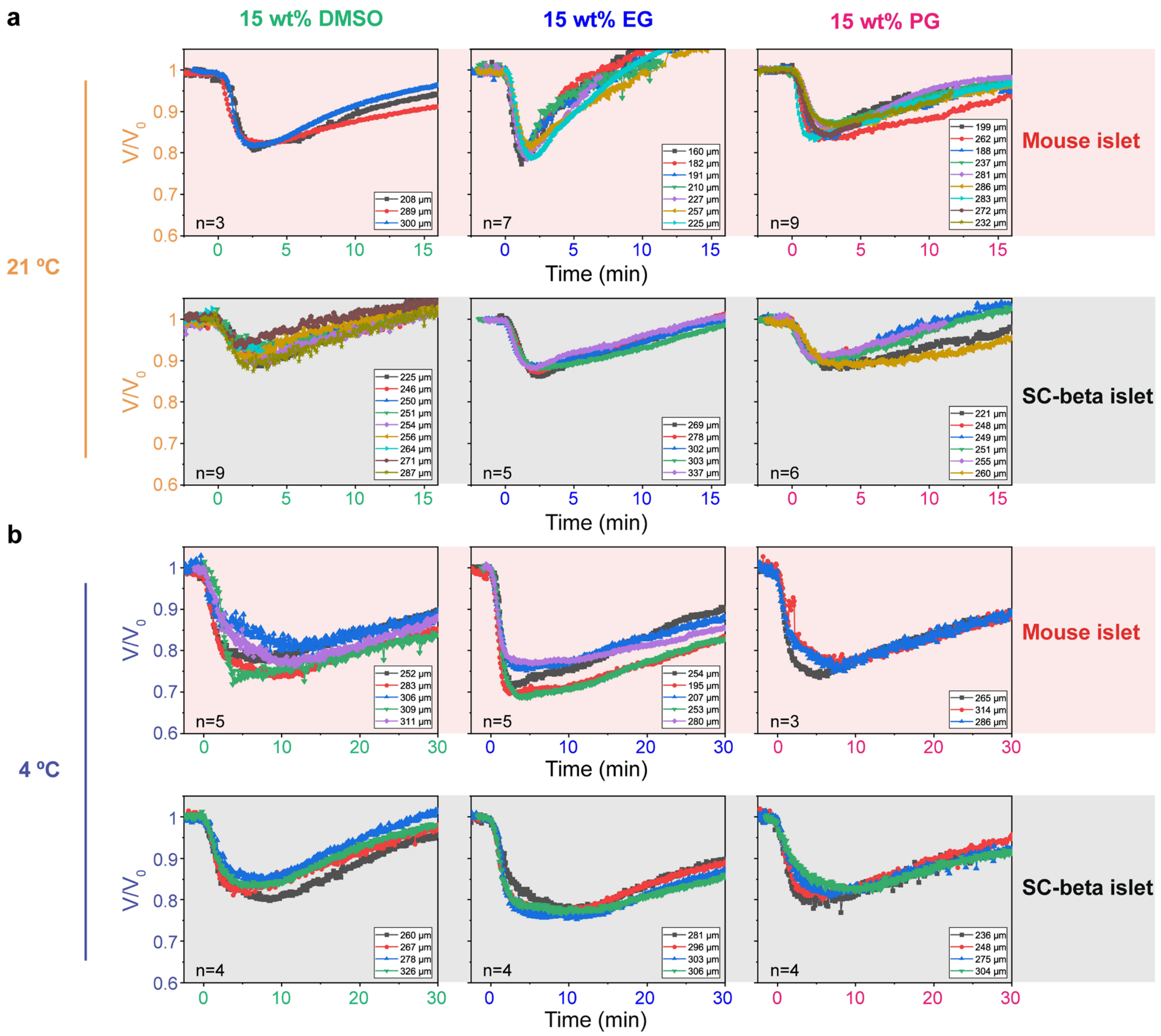

Fig. S3. **Measurement of islet volume change in 15 wt% CPA solutions. a,** At 21°C, the volume response of mouse islet (upper row) and SC-beta islet (lower row) to DMSO (left column), EG (middle column), and PG (right column). **b,** At 4°C, the volume response of mouse islet (upper row) and SC-beta islet (lower row) to DMSO (left column), EG (middle column), and PG (right column). The legends represent the diameter of islets. The number of independent experiment (i.e., n) is included in the plot.

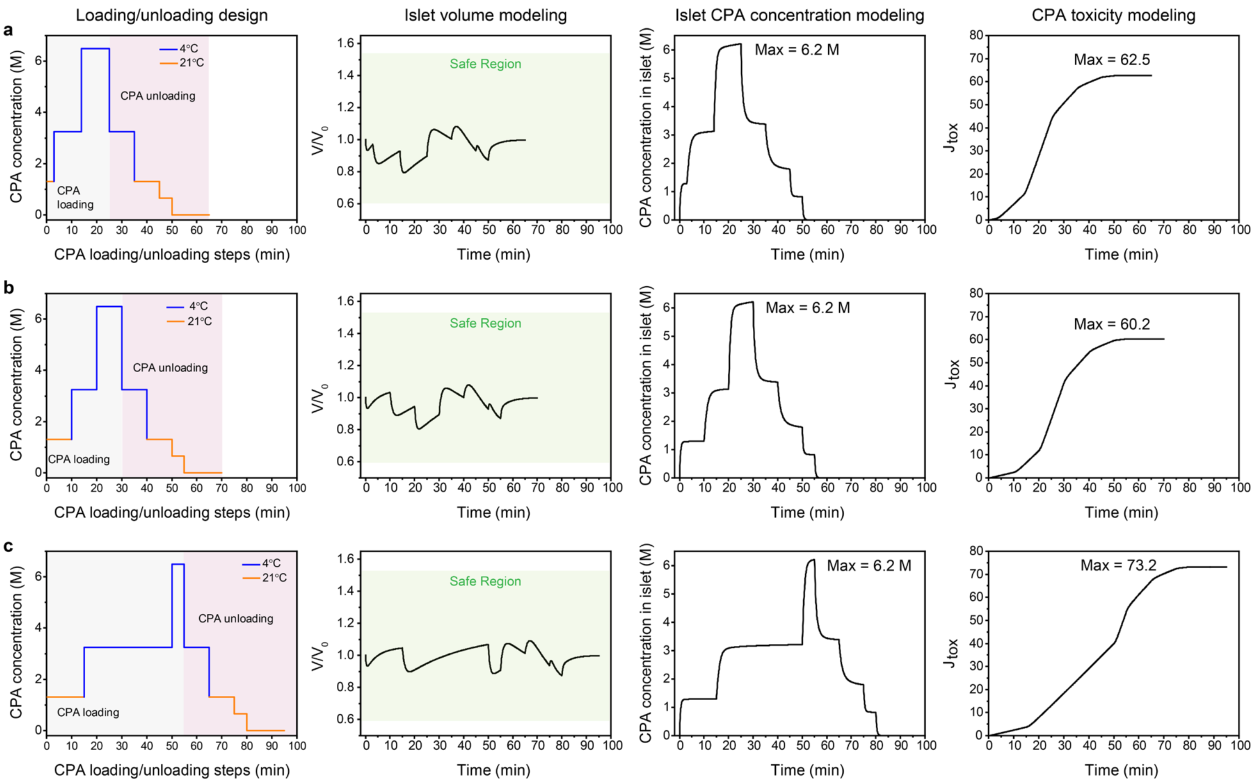

Fig. S4. Modeling of CPA chemical toxicity for short, medium, and long CPA loading step durations. a, For short loading, step durations of 3 min, 11 min, and 11 min were used for the 1.3 M, 3.2 M, and 6.5 M steps, respectively. The final CPA chemical toxicity value (J_{tox}, far right column) was computed to be 62.5. **b,** For medium loading, step durations of 10 min loading time were used for each 1.3 M, 3.2 M, and 6.5 M steps. The final CPA chemical toxicity value (J_{tox}, far right column) was computed to be 60.2. **c,** For long loading, the step durations were 15 min, 35 min, and 5 min for 1.3 M, 3.2 M, and 6.5 M steps, respectively. The final CPA chemical toxicity value (J_{tox}, far right column) was computed to be 73.2. Note that for all 3 loading step approaches (**a-c**), the unloading steps were the same. For all cases, the islet volume was maintained in the safe region (i.e., $0.6 < V/V_0 < 1.53$), and the final islet CPA concentration was 6.2 M. We found the medium loading step duration provided the lowest chemical toxicity value (J_{tox} = 60.2) and was therefore used in our VR protocol.

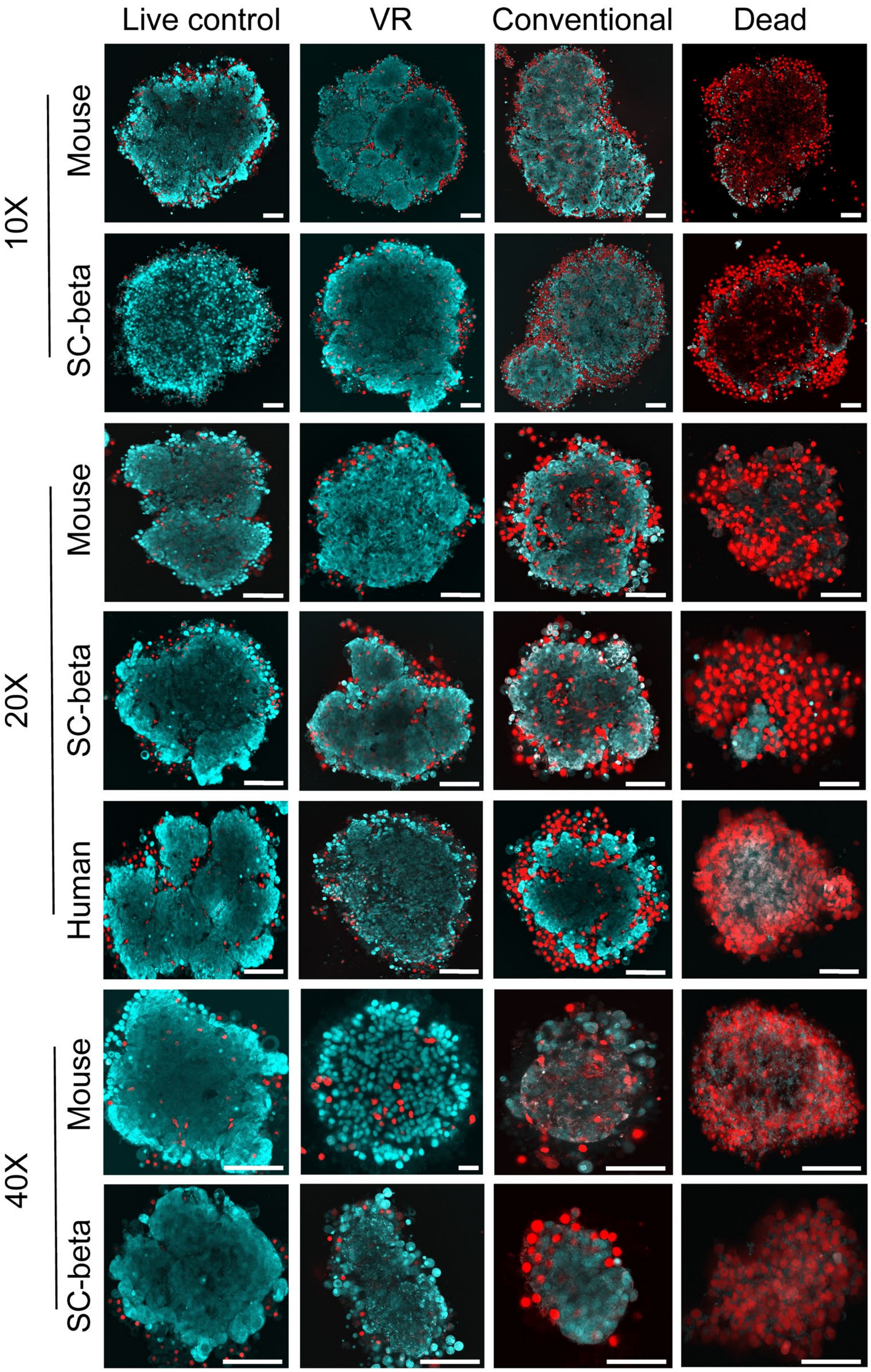

Fig. S5. Confocal images of islets under multiple magnifications. Islets from live control, dead control, conventional cryopreservation and VR cryopreservation were shown as the merged AO/PI staining. Scale bars are 100 µm.

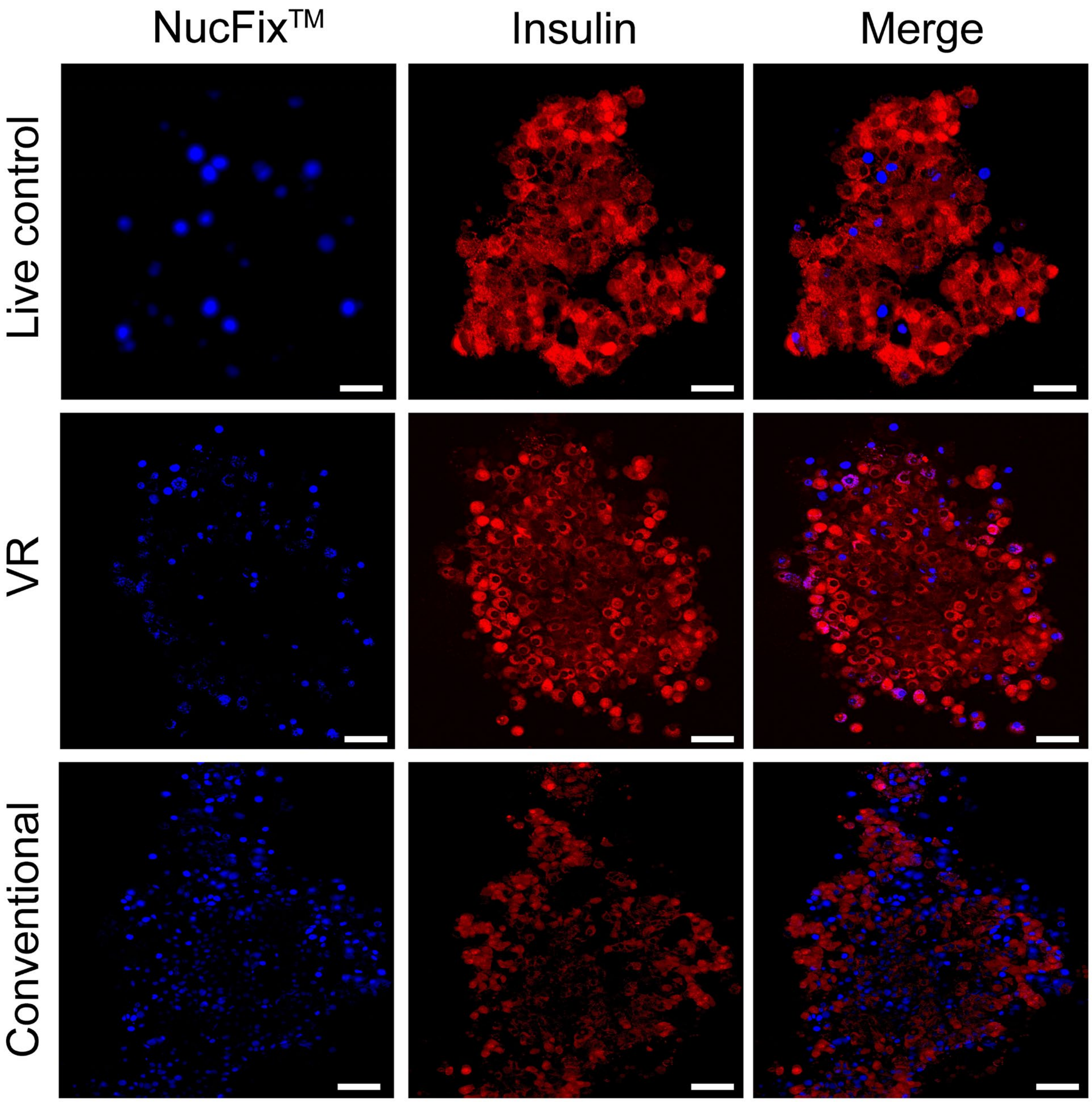

Fig. S6. Mouse beta cell-specific viability. Following treatments indicated, intact mouse islets were stained with fixable live/dead dye (NucFix), fixed, permeabilized and stained with anti-insulin antibodies then imaged by confocal microscopy. NucFix stains the nucleus of dead cells blue, cytoplasmic insulin staining is red. Islets from live control, VR and conventional cryopreservation were displayed. Dead cells (blue nuclei) were present in both beta cell (red cytoplasm) and non-beta cell (dim cytoplasm) populations. Representative images were shown. Scale bars are 100 μm.

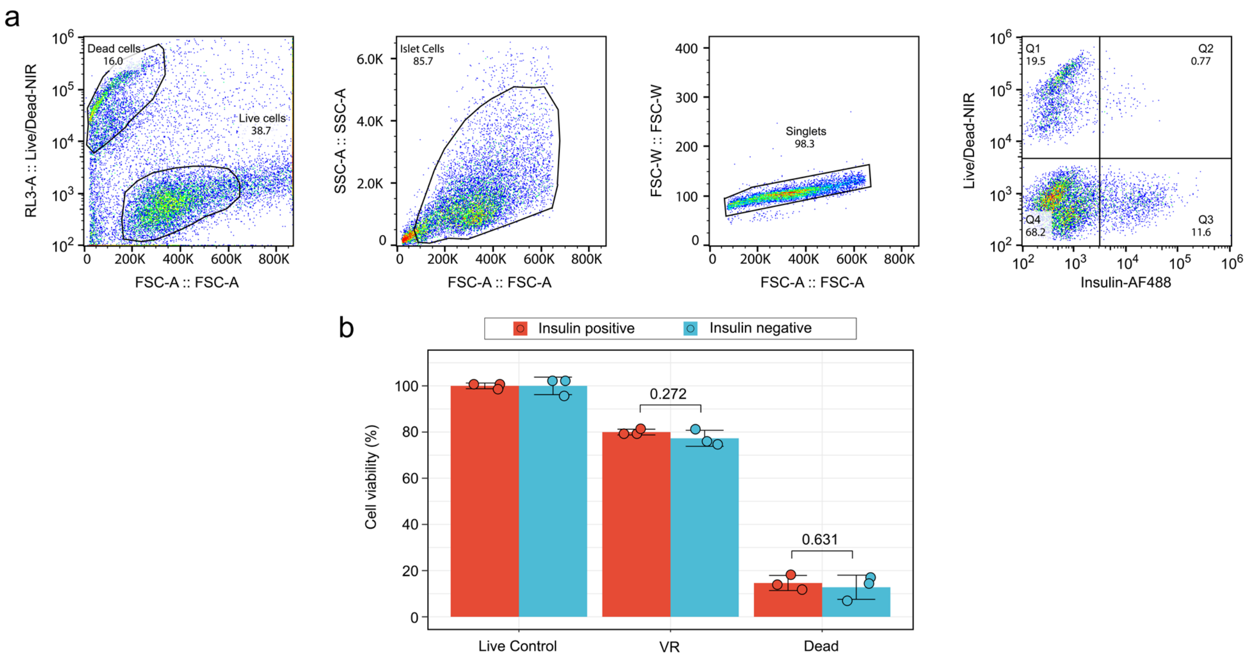

Fig. S7. Beta cell specific viability in SC-beta islets. SC-beta islets were vitrified and rewarmed (VR), then dissociated to single cell suspensions, stained with fixable live/dead dye, fixed, permeabilized, and stained with anti-insulin antibodies. **a,** Representative gating scheme for flow cytometric analysis is shown. An initial live/dead v. forward scatter demonstrated clear populations of live and dead cells. Following FSC/SSC and singlet gating a final 4-quadrant FACS plot showed individual populations of insulin +/- and live/dead cells. **b,** From the FACS plots in a, the percent of live cells in the insulin positive and negative cell populations was determined (normalized relative to the input live control cells). One-way ANOVA with Tukey post hoc test was used to compare groups (n=3/group), and the p values are indicated. Data are individual data points and mean ± s.d.

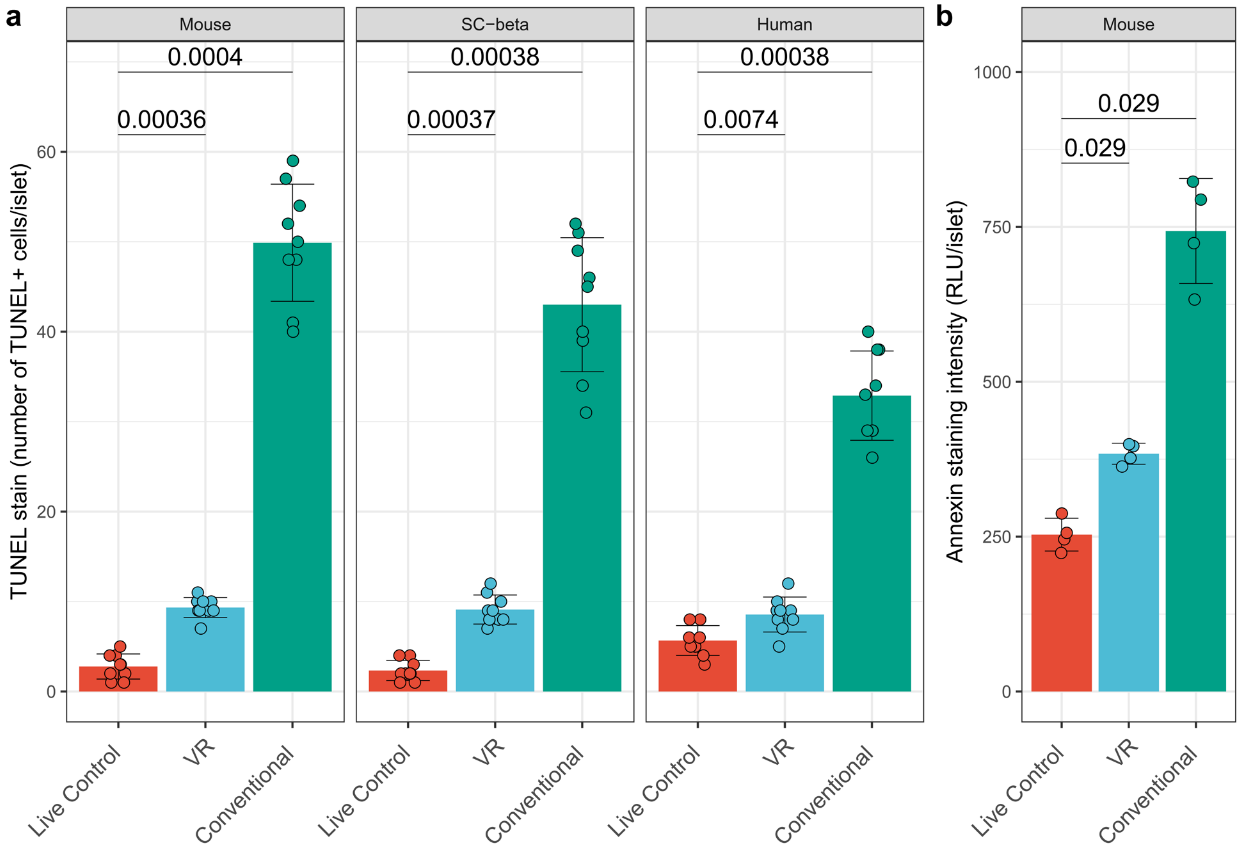

Fig. S8. Quantification of TUNEL and Annexin V translocation. a, The number of TUNEL positive cells per islet was measured for mouse, human, and SC-beta islets following VR vs. conventional cryopreservation and fresh control islets (n = 9/group). **b,** Annexin V translocation to the cell surface was quantified by fluorescence intensity measurement for mouse islets of each treatment group (n = 4/group). Kruskal-Wallis and pairwise Wilcox tests were used to compare groups. Data are individual data points and mean ± s.d.

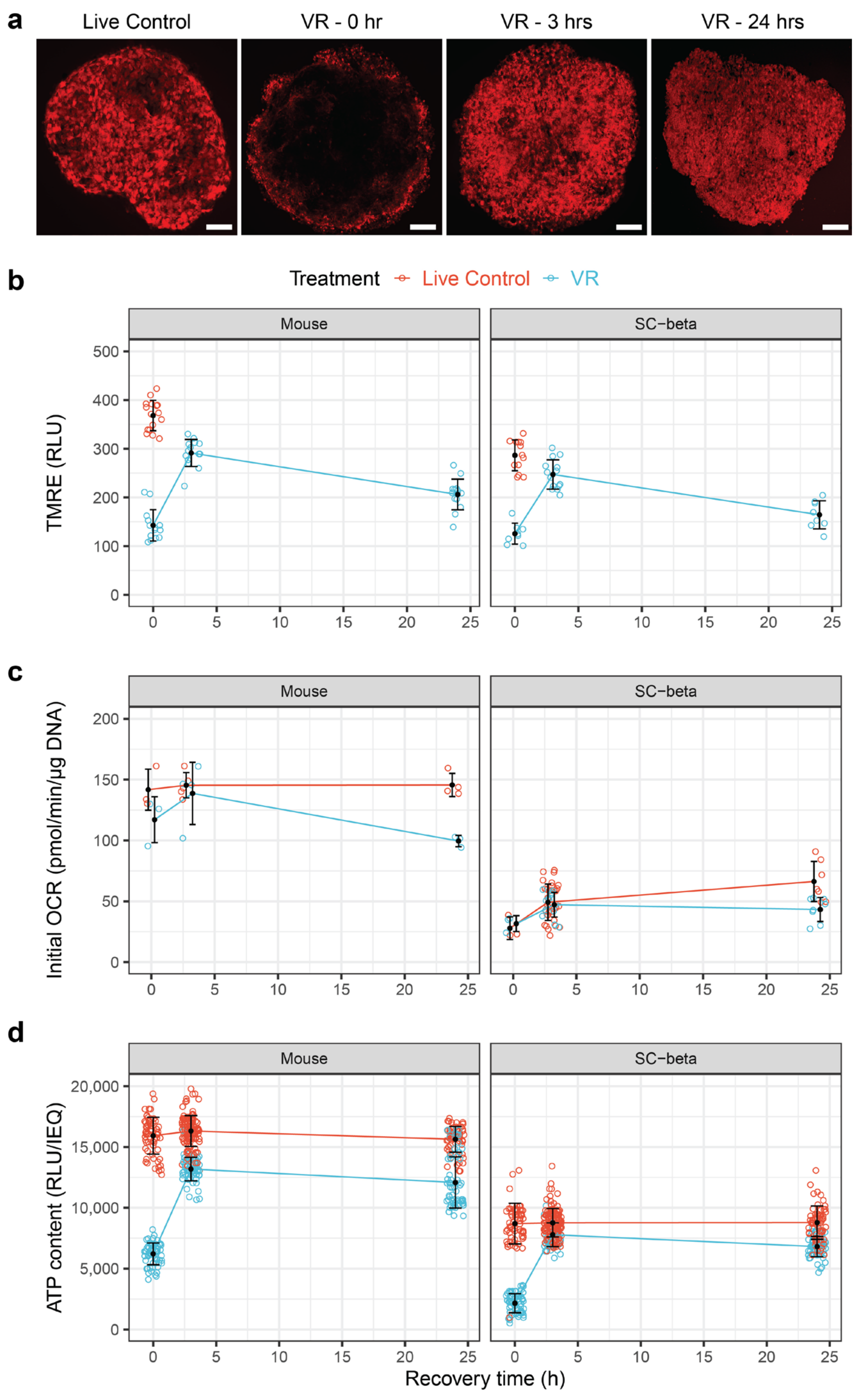

Fig. S9. Mouse and SC-beta islets metabolism recovery post VR. a, Confocal images of TMRE stained mouse islets. From left to right: live control, 0 hr post-VR, 3 hrs post-VR, and 24 hrs post-VR. **b,** Quantitative comparison of TMRE staining intensity from live control, 0 hr post-VR, 3 hrs post-VR, and 24 hrs post-VR islets. **c,** Initial oxygen consumption rate (OCR) of live control, 0 hr post VR, 3 hr post-VR, and 24 hr post-VR islets. **d,** ATP content of live control, 0 hr post-VR, 3 hr post-VR, and 24 hr post VR islets. Error bars are mean ± s.d. Scale bars are 100 µm. For b-d, the exact sample size (n) for each experimental group can be found in Table S3. For this experiment each of the cumulative technical replicates is shown.

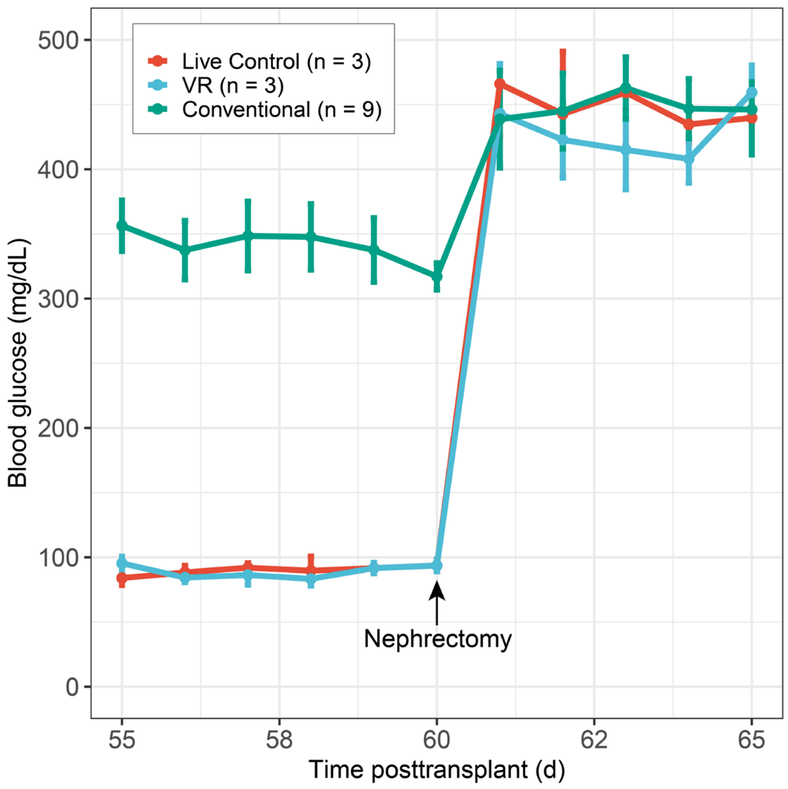

Fig. S10. Blood glucose level before and after nephrectomy. To validate the syngeneic transplant model, a subset of islet transplant recipients underwent nephrectomy of the islet-containing kidney on posttransplant day 60. Blood glucose rise confirmed that the transplanted islets were mainlining glycemic control and not the native pancreas. Data are presented as mean ± s.d.

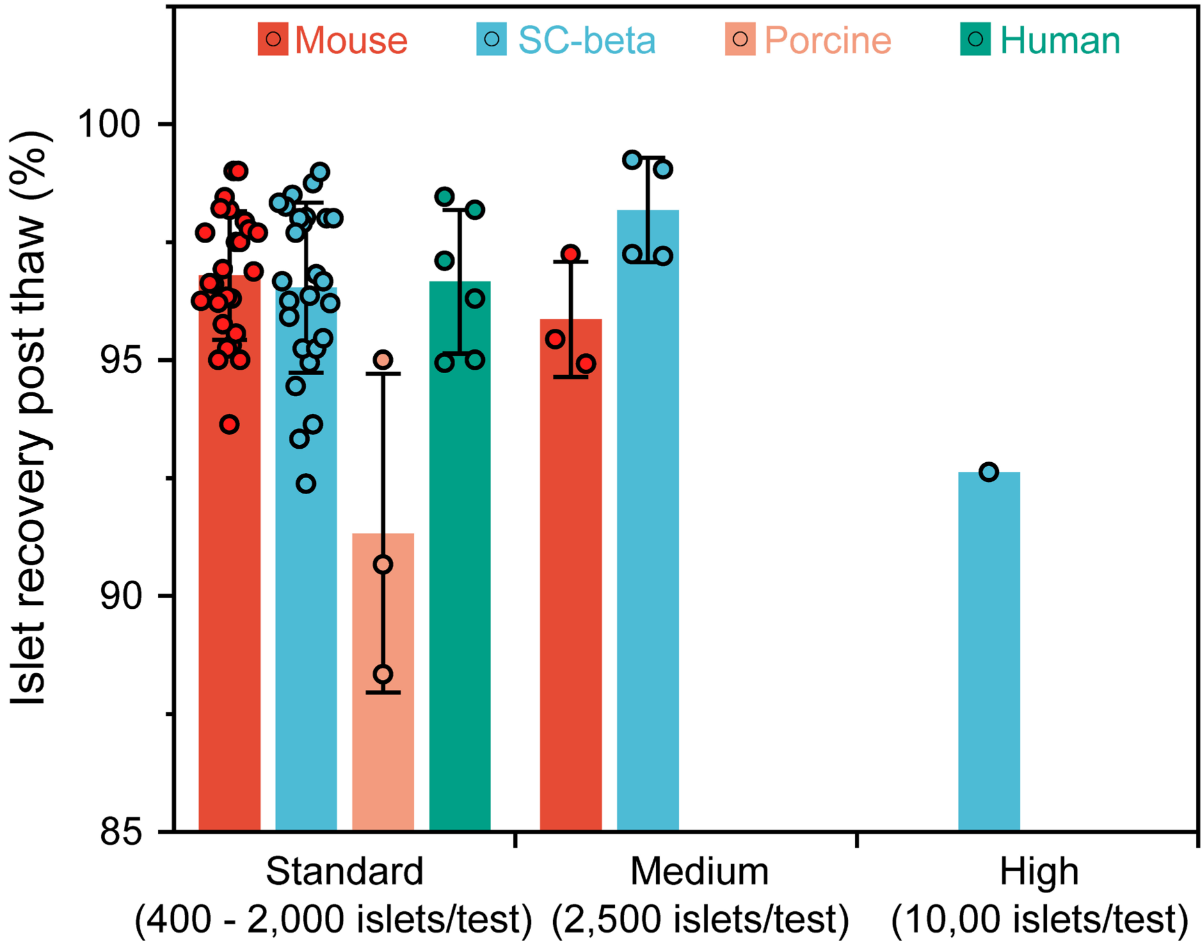

Fig. S11. Recovery of islets post-thaw using VR approach. Islet recovery is the ratio of islet quantity after cryopreservation to islet quantity before cryopreservation. For standard scale, 400 – 2,000 islets were used per test; for medium scale, 2,500 islets were used per test; for high scale, 10,000 islets were used per test. Data are presented as mean ± s.d. with individual measured data points. The number of independent experiments can be found in Table S3.

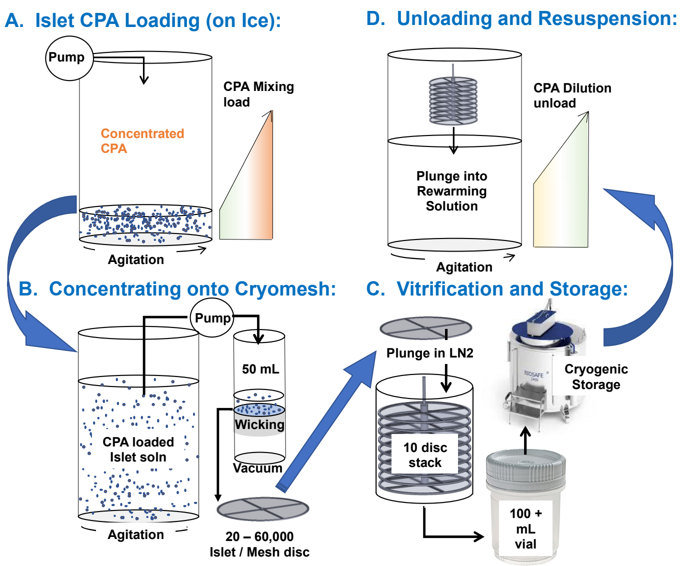

Fig. S12. Conceptual schematic of scaling up. **a.** CPA is loaded in ramp or stepwise fashion while agitating the islet suspension for mixing. For further improvement, a syringe pump could be used to achieve gradual increase of CPA concentration for reduced osmotic injury compared to the stepwise CPA loading used in the current study. **b.** Islets are pumped onto individual cryomesh discs (i.e., ~ 60,000 IEQ/5.5 cm diameter cryomesh disc), and excess CPA is quickly wicked. For example, an estimation of 10 such cryomesh disc would be in the clinically relevant range for transplantation. **c.** Discs are plunged into UV-sterilized LN_2 for vitrification and stored in a sterile, hermetically sealed container in the cryogenic storage freezer. An example of cryomesh stacking for storage is illustrated. **d.** Discs are plunged into rewarming solution, and CPA is offloaded as cells are prepared for transplant. An example of stacked cryomesh for rewarming is displayed.

Table S1. Summary of islets cryopreservation studies

Approach	Cryopreservation details					Post cryopreservation results				Recovery ≥ 90%	Viability ≥ 85%	Function * ≥ 90%	Scalability >1,000 islets	Reference
	Islet quantity	Species	CPA used	Cooling rate (°C/min)	Warming rate (°C/min)	Islets recovery	Viability	GSIS	Transplant					
Conventional (slow freezing)	NR	Rat, Human	0.5 M EG + 1 M DMSO	0.25	~200	80-84%	78-83%	82-94%	Rat islets to nude mice: normoglycemia in 4 days	✗	✗	✓	✗	Kojayan, 2019 [20]
	500	Rat, alginate encapsulated	2 M DMSO	0.25	~200	72-84%	78-88%	82-94%	normoglycemia in 4 days in nude mice	✗	✗	✓	✗	Kojayan, 2019 [21]
	50	Rat	15% DMSO	0.5	~200	NR	50%	28%	NR	✗	✗	✗	✗	Yamanaka, 2016 [22]
	50,000	Human	2 M DMSO	0.25	~200	69%	59%	NR	NR	✗	✗	✗	✗	Miranda, 2013 [23]
	NR	Rat	10 % DMSO	0.25	~200	66%	66%	~60 %	NR	✗	✗	✗	✗	Taylor, 2009 [24]
	100	Rat	1.5 M EG, 5% hydroxy ethylstarlch, 5% trehalose	1	35	85%	NR	94.8%	Normoglycemia in nude mice in 7 days	✗	✗	✓	✗	Miyamoto, 2001 [25]
	500	Mouse	10% DMSO	0.25	~200	80%	NR	NR	Syngeneic transplant, majority achieved normoglycemia	✗	✗	O	✗	Cattral, 1998 [8]
	10,000-35,000	Rat, dog	2M DMSO	0.25	~200	78-92%	NR	33-46%	Canine islets to nude mice: 62% achieved normoglycemia	✗	✗	O	✓	Lakey, 1996 [9]
	NR	Rat, dog	2M DMSO	0.25	200	NR	NR	NR	Normoglycemia delayed by several weeks	✗	✗	O	✗	Rajotte, 1984 [26]
	20	Rat, dog	1M DMSO	variable	7.5	NR	NR	57-100%	NR	✗	✗	O	✗	Bank, 1981 [27]
	NR	Rat	7.5-10% DMSO	0.5-0.7	150	NR	NR	NR	1 rat, 13 weeks to normoglycemia	✗	✗	O	✗	Rajotte, 1977 [28]
Vitrification and Rewarming (VR)	100	Rat	15 % EG + 15% DMSO	NR	NR	94-97%	76%	65%	Normoglycemia in 80 % rat in ~ 3 weeks	✓	✗	O	✗	Nakayama, 2020 [29]
	10-50	Rat	15% EG + 15% DMSO + 0.5 M sucrose	NR	NR	99-100%	64%	38%	NR	✓	✗	✗	✗	Yamanaka 2017 [30]
	25-35	Mouse	15 % EG + 15% DMSO	NR	NR	NR	NR	~ 13%	Normoglycemia in mice in 4-8 days	✗	✗	O	✗	Nagaya 2016 [31]
	10	Rat	15% EG + 15% DMSO + 0.5 M sucrose	23,000	42,000	NR	57%	58%	NR	✗	✗	✗	✗	Yamanaka, 2016 [22]
	100	Rat	30% EG+ 20% DMSO+0.4M trehalose	NR	NR	NR	85.8	91%	Normoglycemia in rats in ~ 2 days	✗	✓	✓	✗	Sasamoto, 2012 [32]
	500	Rat	DP6	43	225	70%	NR	NR	NR	✗	✗	✗	✗	Taylor 2009 [24]
	50	Hamster	5.4M EG + 2 M DMSO	43	200	NR	NR	NR	Normoglycemia in mice in 5 days	✗	✗	O	✗	Agudelo, 2008 [33]
	10,000	Human	20.5% DMSO + 10% PG + 15.5% acetamide	NR	~200	95%	17%	NR	NR	✓	✗	✗	✓	Langer, 1999 [10]
	50-300	Mouse	22.5% DMSO + 11% PG + 17% acetamide	NR	~200	51-61%	65%	~65%	Normoglycemia in mice in several weeks	✗	✗	O	✗	Jutte, 1987 [34]
	50	Human	20.5% DMSO + 10% PG + 15.5% acetamide	NR	~200	80%	85%	44%	NR	✗	✓	✗	✗	Jutte, 1987 [35]
	> 2,500	Mouse, pig, human, SC-beta	22 % EG + 22% DMSO	~59,600	~280,000	95%	Mouse:90.5% Human: 87.4% SC-beta:92.1% Porcine:87.2%	Mouse: 95% Human: 80% SC-beta: 129%	Normoglycemia in 11 out of 12 mice within 1-2 days (250 islets/recipient)	✓	✓	✓	✓	This work

*****, **"✓"** indicates GSIS ≥ 90% and transplant achieves normoglycemia; **"O"** indicates either GSIS ≥ 90% or transplant achieves normoglycemia; **"✗"** indicates GSIS <90% (or NR) and transplant fails to achieve normoglycemia (or NR)

NR: not reported; **CPA**: cryoprotectant agent; **DMSO**: dimethyl sulfoxide; **EG**: ethylene glycol; **PG**: propylene glycol; **GSIS**: glucose-stimulated Insulin Secretion

Table S2. Comparison of Vitrification-Rewarming (VR) approaches

VR approach	Droplet volume (µL)	Cooling rate (×10^4 °C/min)	Warming rate (×10^4 °C/min)	Viability (%)
Copper dish vitrification + convective warming [a]	2	1.1 ± 0.2 (n=5)	1.2 ± 0.2 (n=5)	56 ± 14 (n=5)
Copper dish vitrification + laser nanowarming [b]	2	1.1 ± 0.2 (n=5)	31.3 (by modeling)	62 ± 6.4 (n=5)
Cryotop vitrification + convective warming [c]	2	1.4 ± 0.2 (n=10)	2.3 ± 0.6 (n=10)	55 ± 8.6 (n=4)
Cryomesh vitrification + convective warming [c]	NA	5.3 ± 1.5 (n=6)	30.9 ± 6.5 (n=6)	92.1 ± 1.2 (n=34)

a. Cooling and warming rates are measured using a thermocouple. The thermocouple location is selected to represent the slowest rate inside the droplet (i.e., cooling rate measured at approximately the top of the droplet, warming rate measured at roughly the center of the droplet).
b. The warming rate is the modeling result based on Khosla et al. [20], assuming islet diameter is 150 µm.
c. The cooling and warming rates are measured using a thermocouple at approximately the center of the droplet.

Table S3. Number of experimental replicates and technical replicates for reported results [1]

Figures	Treatment group	Islet sources			
		Mouse islet	SC-beta islet	Porcine islet	Human islet
Figure 2c		4 (1)	8 (1)		
Figure 2d		3 (1)	9 (1)		
Figure 3c-e		4 (3)	4 (3)		
Figure 4b	Live control	34 (3)	45 (3)	3 (3)	4 (3)
	VR	34 (3)	45 (3)	3 (3)	3 (3)
	VR (9 month)	3 (3)	5 (3)		
	Conventional	17 (3)	16 (3)		3 (3)
	Dead control	4 (3)	4 (3)	3 (3)	3 (3)
Figure 4c	Live control	34 (≥4 [3])	45 (≥4 [3])	3 (≥4 [3])	4 (≥4 [3])
	VR	34 (≥4 [3])	45 (≥4 [3])	3 (≥4 [3])	3 (≥4 [3])
	Conventional	17 (≥4 [3])	16 (≥4 [3])		3 (≥4 [3])
	Dead control	4 (≥4 [3])	4 (≥4 [3])	3 (≥4 [3])	3 (≥4 [3])
Figure 4d	TUNEL	6 (5)	10 (4)		3 (5)
	Annexin V	5 (7)			
Figure 5b TMRE	Live control	16 (7)	13 (7)	3 (7)	2 (7)
	VR	14 (7)	14 (7)	3 (7)	3 (7)
	Conventional	9 (7)	5 (7)		3 (7)
	Dead control	4 (7)	4 (7)	3 (7)	3 (7)
Figure 5b ATP	Live control	12 (21)	12 (21)	3 (21)	3 (21)
	VR	3 (21)	3 (21)	3 (21)	3 (21)
	Conventional	3 (21)	3 (21)		3 (21)
	Dead control	12 (9)	12 (9)	3 (9)	3 (9)
Figure 5c	Live control		1 (8)		
	VR		1 (6)		
	Conventional		1 (5)		
	Dead control		1 (5)		
Figure 5d	Live control	5 (4)	33 (5)		4 (3)
	VR	4 (4)	13 (5)		6 (3)
	Conventional	3 (4)	5 (5)		3 (3)
Figure 5e	Live control	12 (3)	4 (3)		3 (3)
	VR	4 (3)	4 (3)		3 (3)
	Conventional	4 (3)	4 (3)		3 (3)
Figure 6c [2]	Live control	10 (1)			
	VR	9 (1)			
	Conventional	10 (1)			
	WT mice	9 (1)			
	Diabetic mice	10 (1)			
Figure 6d [2]	Mock		5 (1)		
	Conventional		3 (1)		
	VR		5 (1)		
Figure 6e [2]	Mock		5 (1)		
	Conventional		3 (1)		
	VR		5 (1)		
Figure S5	Live control	34 (≥4 [3])	45 (≥4 [3])		
	VR	34 (≥4 [3])	45 (≥4 [3])		

	Conventional	17 (≥4 [3])	16 (≥4 [3])		
	Dead control	4 (≥4 [3])	4 (≥4 [3])		
Figure S6	Mock	1 (10)			
	Conventional	1 (10)			
	VR	1 (10)			
Figure S7	Live control		1 (3)		
	VR		1 (3)		
	Conventional		1 (3)		
	Dead		1 (3)		
Figure S8a TUNEL	Live control	1 (9)	1 (9)		1 (9)
	VR	1 (9)	1 (9)		1 (9)
	Conventional	1 (9)	1 (9)		1 (9)
Figure S8b Annexin	Live control	1 (4)			
	VR	1 (4)			
	Conventional	1 (4)			
Figure S10	Live control	3(1)			
	VR	3(1)			
	Conventional	10(1)			
Figure S11	Standard scale	26	26	3	6
	Medium scale	3	4		
	High scale		1		

		Live control			VR		
		0 hr	3 hrs	24 hrs	0 hr	3 hrs	24 hrs
Figure S9b	Mouse islet	16 (7)			14 (7)	14 (7)	14 (7)
	SC-beta islet	13 (7)			8 (7)	14 (7)	8 (7)
Figure S9c	Mouse islet	3 (4)	5 (4)	4 (4)	3 (4)	4 (4)	3 (4)
	SC-beta islet	3 (5)	33 (5)	7 (5)	3 (5)	13 (5)	8 (5)
Figure S9d	Mouse islet	3 (21)	6 (21)	3 (21)	3 (21)	3 (21)	3 (21)
	SC-beta islet	3 (21)	6 (21)	3 (21)	3 (21)	3 (21)	3 (21)

[1] data presented as number of experimental replicates (number of technical replicates in parenthesis). Experimental replicates represent distinct biological samples treated at different days.

[2] unit of measurement is number of mice used.

[3] For confocal imaging the number of experimental replicates was the same as for Fig. 4b (dissociated islet cell viability) and the number of technical replicates (individual islets imaged) was at least 4 islets per condition.

References

1. Liu, Y., *et al.* Photothermal conversion of gold nanoparticles for uniform pulsed laser warming of vitrified biomaterials. *Nanoscale* **12**, 12346-12356 (2020).
2. Zhan, L., *et al.* Conduction Cooling and Plasmonic Heating Dramatically Increase Droplet Vitrification Volumes for Cell Cryopreservation. *Adv Sci (Weinh)* **8**, 2004605 (2021).
3. Zieger, M.A., Woods, E.J., Lakey, J.R., Liu, J. & Critser, J.K. Osmotic tolerance limits of canine pancreatic islets. *Cell transplantation* **8**, 277-284 (1999).
4. Benson, J.D., Benson, C.T. & Critser, J.K. Mathematical model formulation and validation of water and solute transport in whole hamster pancreatic islets. *Mathematical biosciences* **254**, 64-75 (2014).
5. Benson, J.D., Higgins, A.Z., Desai, K. & Eroglu, A. A toxicity cost function approach to optimal CPA equilibration in tissues. *Cryobiology* **80**, 144-155 (2018).
6. Warner, R.M., *et al.* Rapid quantification of multi-cryoprotectant toxicity using an automated liquid handling method. *Cryobiology* **98**, 219-232 (2021).
7. Best, B.P. Cryoprotectant Toxicity: Facts, Issues, and Questions. *Rejuvenation Res* **18**, 422-436 (2015).
8. Cattral, M.S., Lakey, J.R., Warnock, G.L., Kneteman, N.M. & Rajotte, R.V. Effect of cryopreservation on the survival and function of murine islet isografts and allografts. *Cell transplantation* **7**, 373-379 (1998).
9. Lakey, J.R., Warnock, G.L., Ao, Z. & Rajotte, R.V. Bulk cryopreservation of isolated islets of Langerhans. *Cell transplantation* **5**, 395-404 (1996).
10. Langer, S., *et al.* Viability and recovery of frozen-thawed human islets and in vivo quality control by xenotransplantation. *Journal of molecular medicine* **77**, 172-174 (1999).
11. Parmegiani, L., *et al.* Sterilization of liquid nitrogen with ultraviolet irradiation for safe vitrification of human oocytes or embryos. *Fertil Steril* **94**, 1525-1528 (2010).
12. Penzias, A., *et al.* A review of best practices of rapid-cooling vitrification for oocytes and embryos: a committee opinion. *Fertil. Steril.* **115**, 305-310 (2021).
13. Larman, M.G., Hashimoto, S., Morimoto, Y. & Gardner, D.K. Cryopreservation in ART and concerns with contamination during cryobanking. *Reprod. Med. Biol.* **13**, 107-117 (2014).
14. Kedem, O. & Katchalsky, A. Thermodynamic analysis of the permeability of biological membranes to non-electrolytes. *Biochimica et biophysica acta* **27**, 229-246 (1958).
15. Johnson, J.A. & Wilson, T.A. A model for capillary exchange. *The American journal of physiology* **210**, 1299-1303 (1966).
16. McGrath, J.J. Quantitative measurement of cell membrane transport: technology and applications. *Cryobiology* **34**, 315-334 (1997).
17. Mukherjee, I.N., Song, Y.C. & Sambanis, A. Cryoprotectant delivery and removal from murine insulinomas at vitrification-relevant concentrations. *Cryobiology* **55**, 10-18 (2007).
18. Benson, J.D., Kearsley, A.J. & Higgins, A.Z. Mathematical optimization of procedures for cryoprotectant equilibration using a toxicity cost function. *Cryobiology* **64**, 144-151 (2012).
19. Han, Z. & Bishop, J.C. PERSPECTIVE: Critical Cooling and Warming Rates as a Function of CPA Concentration. *Cryo letters* **41**, 185-193 (2020).
20 Kojayan, G. *et al.* Improved cryopreservation yield of pancreatic islets using combination of lower dose permeable cryoprotective agents. *Cryobiology* **88**, 23-28 (2019).
21 Kojayan, G. G., Flores, A., Li, S., Alexander, M. & Lakey, J. R. Cryopreserved alginate-encapsulated islets can restore Euglycemia in a diabetic animal model better than cryopreserved non-encapsulated islets. *Cell Med.* **11**, 2155179019876641 (2019).
22 Yamanaka, T. *et al.* Direct comparison of Cryotop® vitrification and Bicell® freezing on recovery of functional rat pancreatic islets. *Cryobiology* **73**, 376-382 (2016).
23 Miranda, P. M. *et al.* Human islet mass, morphology, and survival after cryopreservation using the Edmonton protocol. *Islets* **5**, 188-195 (2013).

24 Taylor, M. J. & Baicu, S. Review of vitreous islet cryopreservation: Some practical issues and their resolution. *Organogenesis* **5**, 155-166 (2009).
25 Miyamoto, M. *et al.* Development of a cryopreservation procedure employing a freezer bag for pancreatic islets using a newly developed cryoprotectant. *Cell transplant.* **10**, 363-371 (2001).
26 Rajotte, R. V., Warnock, G. L. & Kneteman, N. N. Cryopreservation of insulin-producing tissue in rats and dogs. *World J. Surg.* **8**, 179-186 (1984).
27 Bank, H. L. & Reichard, L. Cryogenic preservation of isolated islets of Langerhans: Two-step cooling. *Cryobiology* **18**, 489-496 (1981).
28 Rajotte, R., Stewart, H., Voss, W., Shnitka, T. & Dossetor, J. Viability studies on frozen-thawed rat islets of Langerhans. *Cryobiology* **14**, 116-120 (1977).
29 Nakayama-Iwatsuki, K. *et al.* Transplantation of rat pancreatic islets vitrified-warmed on the nylon mesh device and the silk fibroin sponge disc. *Islets* **12**, 145-155 (2020).
30 Yamanaka, T., Goto, T., Hirabayashi, M. & Hochi, S. Nylon mesh device for vitrification of large quantities of rat pancreatic islets. *Biopreserv. Biobank.* **15**, 457-462 (2017).
31 Nagaya, M. *et al.* An effective new cryopreservation procedure for pancreatic islets using hollow fiber vitrification. *Horm. Metab. Res* **48**, 540-549 (2016).
32 Sasamoto, H., Futami, M., Ando, Y. & Nakaji, S. Cryopreservation of rat islets of Langerhans by vitrification. *J. Artf. Organs* **15**, 283-289 (2012).
33 Agudelo, C. A. & Iwata, H. The development of alternative vitrification solutions for microencapsulated islets. *Biomaterials* **29**, 1167-1176 (2008).
34 Jutte, N., Heyse, P., Jansen, H., Bruining, G. & Zeilmaker, G. Vitrification of mouse islets of Langerhans: comparison with a more conventional freezing method. *Cryobiology* **24**, 292-302 (1987).
35 Jutte, N., Heyse, P., Jansen, H., Bruining, G. & Zeilmaker, G. Vitrification of human islets of Langerhans. *Cryobiology* **24**, 403-411 (1987).
