# Supplementary material for: Pancreatic islet cryopreservation by vitrification achieves high viability, function, recovery and clinical scalability for transplantation
Source: Nat Med. 2022 Mar 14;28(4):798–808. doi: 10.1038/s41591-022-01718-1 (PMC9018423; doi:10.1038/s41591-022-01718-1)
Supplement: Supplementary file 2 — Reporting Summary [file 41591_2022_1718_MOESM2_ESM.pdf]

nature portfolio

Corresponding author(s): Erik Finger

Last updated by author(s): 1/18/2022

Reporting Summary

Nature Portfolio wishes to improve the reproducibility of the work that we publish. This form provides structure for consistency and transparency in reporting. For further information on Nature Portfolio policies, see our Editorial Policies and the Editorial Policy Checklist.

Statistics

For all statistical analyses, confirm that the following items are present in the figure legend, table legend, main text, or Methods section.

n/a	Confirmed	
☐	☒	The exact sample size (n) for each experimental group/condition, given as a discrete number and unit of measurement
☐	☒	A statement on whether measurements were taken from distinct samples or whether the same sample was measured repeatedly
☐	☒	The statistical test(s) used AND whether they are one- or two-sided<br>*Only common tests should be described solely by name; describe more complex techniques in the Methods section.*
☒	☐	A description of all covariates tested
☐	☒	A description of any assumptions or corrections, such as tests of normality and adjustment for multiple comparisons
☐	☒	A full description of the statistical parameters including central tendency (e.g. means) or other basic estimates (e.g. regression coefficient) AND variation (e.g. standard deviation) or associated estimates of uncertainty (e.g. confidence intervals)
☐	☒	For null hypothesis testing, the test statistic (e.g. F, t, r) with confidence intervals, effect sizes, degrees of freedom and P value noted<br>*Give P values as exact values whenever suitable.*
☒	☐	For Bayesian analysis, information on the choice of priors and Markov chain Monte Carlo settings
☒	☐	For hierarchical and complex designs, identification of the appropriate level for tests and full reporting of outcomes
☒	☐	Estimates of effect sizes (e.g. Cohen's d, Pearson's r), indicating how they were calculated

Our web collection on statistics for biologists contains articles on many of the points above.

Software and code

Policy information about availability of computer code

Data collection	The following softwares were used for data collection: MATLAB 2018b, Olympus CellSens Dimension software (v1.17), BioTek Gen5, NXT v. 3.1 (ThermoFisher)
Data analysis	The following softwares were used for data analysis: R 4.0.3, MATLAB 2019a, Olympus CellSens Dimension software (v1.17), BioTek Gen5, and FlowJo v.10 (FlowJo, Ashland, OR)

For manuscripts utilizing custom algorithms or software that are central to the research but not yet described in published literature, software must be made available to editors and reviewers. We strongly encourage code deposition in a community repository (e.g. GitHub). See the Nature Portfolio guidelines for submitting code & software for further information.

Data

Policy information about availability of data

All manuscripts must include a data availability statement. This statement should provide the following information, where applicable:

- Accession codes, unique identifiers, or web links for publicly available datasets
- A description of any restrictions on data availability
- For clinical datasets or third party data, please ensure that the statement adheres to our policy

All data are deposited in the Data Repository of the University of Minnesota (DRUM) and are freely available.

Field-specific reporting

Please select the one below that is the best fit for your research. If you are not sure, read the appropriate sections before making your selection.

☒ Life sciences ☐ Behavioural & social sciences ☐ Ecological, evolutionary & environmental sciences

For a reference copy of the document with all sections, see nature.com/documents/nr-reporting-summary-flat.pdf

Life sciences study design

All studies must disclose on these points even when the disclosure is negative.

Sample size

Based on initial viability testing in mouse islets the normalized per cell viability was 100 ± 2.4% for control islets and 90.5 ± 2.6% for treated (vitrified and rewarmed). Using standard assumptions (alpha = 0.05, beta = 0.2, equal distribution between groups), we use the T statistic and determined the minimum sample size in each group (n = 3). However, since not all test parameters examined were normally distributed, we would need a group size of $n \geq 4$ to be able to compare continuous variables with the possibility of demonstrating difference in the mean at a probability $p < 0.05$ for non-parametric tests. Thus, minimum sample size for statistical comparisons in this study were ≥ 3 for normally distributed parameters and ≥ 4 for non-normal.

Data exclusions

No data was excluded from the analysis

Replication

The number of replication in each experiment was represented by the number of data points in the presented figures. More than 3 replicates were performed in each experiment. All our attempts at replication were successful.

Randomization

Our sample were allocated randomly

Blinding

For different treatment groups, the islets were randomly selected and the outcome is determined by direct imaging and/or commercial readout devices. Wherever possible the outcome measures were determined by automated (i.e. computational) routine to minimize or avoid observational bias. Subjective observations (ie, gross appearance of islets) were clearly described as such in the text.

Reporting for specific materials, systems and methods

We require information from authors about some types of materials, experimental systems and methods used in many studies. Here, indicate whether each material, system or method listed is relevant to your study. If you are not sure if a list item applies to your research, read the appropriate section before selecting a response.

Materials & experimental systems

n/a	Involved in the study	
☐	☒	Antibodies
☐	☒	Eukaryotic cell lines
☒	☐	Palaeontology and archaeology
☐	☒	Animals and other organisms
☒	☐	Human research participants
☒	☐	Clinical data
☒	☐	Dual use research of concern

Methods

n/a	Involved in the study	
☒	☐	ChIP-seq
☐	☒	Flow cytometry
☒	☐	MRI-based neuroimaging

Antibodies

Antibodies used

FLEX Polyclonal Guinea Pig Anti-Insulin Antibody (Agilent; Cat # IR002)
Rabbit Anti-Glucagon antibody (abcam; Cat # ab92517)
Goat anti-Guinea Pig IgG (H+L) Secondary Antibody (Alexa® Flour 488) (abcam; Cat # ab150185)
Goat Anti-Rabbit IgG H&L (Alexa® Flour 647) (abcam; Cat # ab150079)
Mouse Monoclonal Anti-Insulin antibody [clone K36aC10] (abcam; Cat # ab6995)
Goat Anti-Mouse IgG H&L (Alexa Flour® 647) (Abcam; Cat # ab150115)
Goat Anti-Rabbit IgG H&L (Alexa® Flour 488) (abcam; Cat # ab150077)
goat anti-PDX1 (R&D Systems, AF2419)
mouse anti-NKX6.1 (DSHB, F55A12),
rabbit anti-chromogranin (Abcam, ab15160)
rat anti- c-peptide (DSHB, GN-ID4)
mouse anti-glucagon (Sigma, G2654).
donkey anti-goat Alexa Fluor 488 (Invitrogen, A-11055, 1:1,000)
donkey anti-rabbit Alexa Fluor 488 (Invitrogen, A-21206, 1:1,000)
donkey anti-rat Alexa Fluor 488 (Invitrogen, A-21208, 1:1,000)
donkey-anti-mouse-Alexa Fluor 647 (A-31571, 1:1,000)

Validation

FLEX Polyclonal Guinea Pig Anti-Insulin Antibody (Agilent; Cat # IR002)
Validation: “The antibody cross-reacts with insulin from several mammalian species. Specificity as determined by radioimmunoassay was 100% for human insulin, 100% for porcine insulin and less than 0.05% for glucagon and human growth hormone. This product has been optimized for use on human tissues.”

Rabbit Anti-Glucagon antibody (abcam; Cat # ab92517)
Validation: IHC-P: Murine, human, and rat pancreatic tissue. IHC-Fr: Mouse pancreas tissue. WB: Capan-1 cell lysate, Mouse and rat pancreas tissue lysate
Publications (of 43 listed):
• Zhou L et al. Induced regulatory T cells suppress Tc1 cells through TGF-ß signaling to ameliorate STZ-induced type 1 diabetes mellitus. Cell Mol Immunol 18:698-710 (2021).PubMed: 33446887
• Wang MY et al. Glucagon blockade restores functional ß-cell mass in type 1 diabetic mice and enhances function of human islets. Proc Natl Acad Sci U S A 118:N/A (2021).PubMed: 33619103
• Kim GS et al. Optimal allogeneic islet dose for transplantation in insulin-dependent diabetic Macaca fascicularis monkeys. Sci Rep 11:8617 (2021).PubMed: 33883656
• Mimouni NEH et al. Polycystic ovary syndrome is transmitted via a transgenerational epigenetic process. Cell Metab 33:513-530.e8 (2021).PubMed: 33539777
• Yang X et al. Coregulator Sin3a Promotes Postnatal Murine ß-Cell Fitness by Regulating Genes in Ca2+ Homeostasis, Cell Survival, Vesicle Biosynthesis, Glucose Metabolism, and Stress Response. Diabetes 69:1219-1231 (2020).

Goat anti-Guinea Pig IgG (H+L) Secondary Antibody (Alexa® Flour 488) (abcam; Cat # ab150185)
Validation (example): “ICC/IF image of ab7291 stained HeLa cells. The cells were 100% methanol fixed (5 min) and then incubated in 1%BSA / 0.3M glycine in 0.1% PBS-Tween for 1h to permeabilise the cells and block non-specific protein-protein interactions. The cells were then incubated with the antibody (ab7291, 1µg/ml) overnight at +4°C. The cells were then incubated with ab7075, rabbit anti-mouse HRP conjugated antibody, for 1h at a 1/250 dilution. The cells were then incubated with ab34580, guinea pig anti-HRP antibody, for 1h at a 1/250 dilution. Lastly, the secondary antibody (green) was ab150185, polyclonal Secondary Antibody to Guinea pig IgG - H&L (Alexa Fluor® 488), used at 1µg/ml for 1h, as a quaternary antibody. DAPI was used to stain the cell nuclei (blue) at a concentration of 1.43µM.”

References (of 25 listed):
• Almacellas E et al. Lysosomal degradation ensures accurate chromosomal segregation to prevent chromosomal instability. Autophagy 17:796-813 (2021).PubMed: 32573315
• Turecek J & Regehr WG Cerebellar and vestibular nuclear synapses in the inferior olive have distinct release kinetics and neurotransmitters. Elife 9:N/A (2020).PubMed: 33259288
• Matsunari H et al. Compensation of Disabled Organogeneses in Genetically Modified Pig Fetuses by Blastocyst Complementation. Stem Cell Reports 14:21-33 (2020).PubMed: 31883918
• Kurashige T et al. Hormonal Regulation of Autophagy in Thyroid PCCL3 Cells and the Thyroids of Male Mice. J Endocr Soc 4:bvaa054 (2020).PubMed: 32671315
• Zhou Y et al. RILP Restricts Insulin Secretion Through Mediating Lysosomal Degradation of Proinsulin. Diabetes 69:67-82 (2020).

Goat Anti-Rabbit IgG H&L (Alexa® Flour 647) (abcam; Cat # ab150079)
Validation: “ICC/IF image of ab6046 in HeLa cells. The cells were 100% methanol fixed (5 min), permeabilized with 0.1% Triton X-100 for 5 minutes and then incubated in 1%BSA / 10% normal goat serum / 0.3M glycine in 0.1% PBS-Tween for 1h to block non-specific protein-protein interactions. The cells were then incubated with the antibody (ab6046, 2µg/ml) overnight at +4°C. The secondary antibody ab150079 (shown in red) was used at 1µg/ml for 1h. DAPI was used to stain the cell nuclei (blue) at a concentration of 1.43µM. The negative control (inset) is a secondary-only assay to demonstrate low non-specific binding of the secondary antibody.”
“Cross-reactivity of the polyclonal secondary antibody ab182016 was tested using a sandwich ELISA approach. The wells were coated with the indicated IgG standards at 1 µg/ml (50 µl/well) and incubated overnight at 4°C, followed by a 5% BSA blocking step for 2h at RT. ab182016 was then added starting at 1 µg/ml and gradually diluted 1/4 (50 µl/well), followed by incubation for 2h. For the detection Donkey anti-Goat IgG H&L (HRP) (ab6885) was used at 1/10,000 dilution (50 µl/well), followed by incubation for 1h at RT.”
“Cross-reactivity of Goat anti-Rabbit IgG H&L (ab182016) and Goat anti-Rabbit IgG H&L obtained from two different vendors was tested using a sandwich ELISA approach. The wells were coated with the indicated IgG standards (Rabbit, Human, Mouse and Rat) at 1 µg/ml (50 µl/well) and incubated overnight at 4°C, followed by a 5% BSA blocking step for 2h at RT. Secondary antibodies were then added starting at 1 µg/ml and gradually diluted 1/4 (50 µl/well), followed by incubation for 2h. For the detection Donkey anti-Goat IgG H&L (HRP) (ab6885) was used at 1/10,000 dilution (50 µl/well), followed by incubation for 1h at RT.”

References (of 176 listed):
• Wu F et al. Bleomycin A5 suppresses Drp1-mediated mitochondrial fission and induces apoptosis in human nasal polyp-derived fibroblasts. Int J Mol Med 47:346-360 (2021).PubMed: 33236140
• Daly AC et al. 3D bioprinting of high cell-density heterogeneous tissue models through spheroid fusion within self-healing hydrogels. Nat Commun 12:753 (2021).PubMed: 33531489
• Qian F et al. MiR-378a-3p as a putative biomarker for hepatocellular carcinoma diagnosis and prognosis: Computational screening with experimental validation. Clin Transl Med 11:e307 (2021).PubMed: 33634974
• Toro CA et al. The Human ApoE4 Variant Reduces Functional Recovery and Neuronal Sprouting After Incomplete Spinal Cord Injury in Male Mice. Front Cell Neurosci 15:626192 (2021).PubMed: 33679326
• Yao LC et al. Four potential microRNAs affect the progression of pancreatic ductal adenocarcinoma by targeting MET via the PI3K/AKT signaling pathway. Oncol Lett 21:326 (2021).

Mouse Monoclonal Anti-Insulin antibody [K36aC10] (abcam; Cat # ab6995)
Validation: “The antibody exhibits cross-reactivity with human proinsulin. This antibody recognizes purified insulin from the pancreas of human, bovine, horse, sheep, and proinsulin from human. Cross reaction has been observed with insulin containing cells in fixed sections of pancreas from human, porcine, dog, rabbit, bovine, sheep, rat, guinea pig and cat.”
References (of 58 listed):

• Graham GV et al. Effects of long-acting analogues of lamprey GLP-1 and paddlefish glucagon on alpha- to beta-cell transdifferentiation in an insulin-deficient transgenic mouse model. J Pept Sci 27:e3328 (2021).PubMed: 33843129
• Kim GS et al. Optimal allogeneic islet dose for transplantation in insulin-dependent diabetic Macaca fascicularis monkeys. Sci Rep 11:8617 (2021).PubMed: 33883656
• Lafferty RA et al. Positive Effects of NPY1 Receptor Activation on Islet Structure Are Driven by Pancreatic Alpha- and Beta-Cell Transdifferentiation in Diabetic Mice. Front Endocrinol (Lausanne) 12:633625 (2021).PubMed: 33716983
• Moroki T et al. Databases for technical aspects of immunohistochemistry: 2021 update. J Toxicol Pathol 34:161-180 (2021).PubMed: 33976473
• Weng Q et al. STAT3 dictates ß-cell apoptosis by modulating PTEN in streptozocin-induced hyperglycemia. Cell Death Differ 27:130-145 (2020).

Goat Anti-Mouse IgG H&L (Alexa Flour® 647) (Abcam; Cat # ab150115)
Validation: “Specificity: ab150115 is specific to Mouse IgG. ab150115 has less than 47% cross-reactivity with rat IgG. Tested applications: Suitable for: IHC-Fr, ICC/IF, ELISA, IHC-P, Flow Cytometry”

References (from 197 listed):
• Lee SG et al. ?-Tocotrienol-Loaded Liposomes for Radioprotection from Hematopoietic Side Effects Caused by Radiotherapeutic Drugs. J Nucl Med 62:584-590 (2021).PubMed: 32826318
• Li Y et al. Songorine promotes cardiac mitochondrial biogenesis via Nrf2 induction during sepsis. Redox Biol 38:101771 (2021).PubMed: 33189984
• Li C et al. Changes in the expression of endothelial monocyte-activating polypeptide II in the rat hippocampus following status epilepticus. Int J Mol Med 47:699-707 (2021).PubMed: 33416103
• Zou W et al. ASK1/p38-mediated NLRP3 inflammasome signaling pathway contributes to aberrant retinal angiogenesis in diabetic retinopathy. Int J Mol Med 47:732-740 (2021).PubMed: 33416127
• Yang G & Zhao Y MicroRNA-490-3p inhibits inflammatory responses in LPS-induced acute lung injury of neonatal rats by suppressing the IRAK1/TRAF6 pathway. Exp Ther Med 21:152 (2021).

Goat Anti-Rabbit IgG H&L (Alexa® Flour 488) (abcam; Cat # ab150077)
Validation (example): “ICC/IF image of beta Tubulin staining in HeLa cells. The cells were 100% methanol fixed (5 min), permeabilized with 0.1% Triton X-100 for 5 minutes and then incubated in 1%BSA / 10% normal goat serum / 0.3M glycine in 0.1% PBS-Tween for 1h to block non-specific protein-protein interactions. The cells were then incubated with the primary antibody (ab6046, 5µg/ml) overnight at +4°C. The secondary antibody (green) was ab150077 Alexa Fluor® 488 goat anti-rabbit IgG (H+L) used at 2µg/ml for 1h. DAPI was used to stain the cell nuclei (blue) at a concentration of 1.43µM. The negative control (inset) is a secondary-only assay to demonstrate low non-specific binding of the secondary antibody.”

References (from 1255 listed):
• Xiao M et al. Long non-coding RNA H19 promotes the proliferation, migration and invasion while inhibits apoptosis of hypertrophic scarring fibroblasts by targeting miR-3187-3p/GAB1 axis. Burns 47:654-664 (2021).PubMed: 32888745
• Gan L et al. Mesenchymal stem cells promote chemoresistance by activating autophagy in intrahepatic cholangiocarcinoma. Oncol Rep 45:107-118 (2021).PubMed: 33155663
• Li Y et al. Songorine promotes cardiac mitochondrial biogenesis via Nrf2 induction during sepsis. Redox Biol 38:101771 (2021).PubMed: 33189984
• Wei H et al. miR-34c-5p targets Notch1 and suppresses the metastasis and invasion of cervical cancer. Mol Med Rep 23:N/A (2021).PubMed: 33300051
• Niu YT et al. In the presence of TGF-ß1, Asperosaponin VI promotes human mesenchymal stem cell differentiation into nucleus pulposus like- cells. BMC Complement Med Ther 21:32 (2021).

goat anti-PDX1 (R&D Systems, AF2419)
Validation: “Detects human PDX 1/IPF1 in direct ELISAs and Western blots. In direct ELISAs, approximately 45% cross-reactivity with recombinant mouse PDX-1 is observed.”

References (from 25 listed):
• Single-Cell Transcriptome Profiling Reveals &beta Cell Maturation in Stem Cell-Derived Islets after Transplantation. P Augsornwor, KG Maxwell, L Velazco-Cr, JR Millman. Cell Rep, 2020;32(8):108067.
• FGF2 Inhibits Early Pancreatic Lineage Specification during Differentiation of Human Embryonic Stem Cells. Authors: R Dettmer, K Cirksena, J Münchhoff, J Kresse, U Diekmann, I Niwolik, FFR Buettner, O Naujok. Cells, 2020;9(9).
• Targeting the cytoskeleton to direct pancreatic differentiation of human pluripotent stem cells. NJ Hogrebe, P Augsornwor, KG Maxwell, L Velazco-Cr, JR Millman. Nat. Biotechnol., 2020.
• HIV-1-induced cytokines deplete homeostatic innate lymphoid cells and expand TCF7-dependent memory NK cells. Y Wang, L Lifshitz, K Gellatly, CL Vinton, K Busman-Sah, S McCauley, P Vangala, K Kim, A Derr, S Jaiswal, A Kucukural, P McDonel, PW Hunt, T Greenough, J Houghton, M Somsouk, JD Estes, JM Brenchley, M Garber, SG Deeks, J Luban. Nat. Immunol., 2020;21(3):274-286.

mouse anti-NKX6.1 (DSHB, F55A12),
Validation: “Positive Tested Species Reactivity: Bovine, Human, Mouse, Porcine, Rat”

References (of 13 listed):
• Immunohistochemistry of pancreatic development in cattle and pig. Hyttel P. Anatomia, histologia, embryologia 39.2 (2010 Apr): 107-19.
• PCP effector proteins inturned and fuzzy play nonredundant roles in the patterning but not convergent extension of mammalian neural tube. Liu A. Developmental dynamics : an official publication of the American Association of Anatomists 240.8 (2011 Aug): 1938-48.

• Long-term persistence and development of induced pancreatic beta cells generated by lineage conversion of acinar cells. Zhou Q. Nature biotechnology 32.12 (2014 Dec): 1223-30.
• Matched miRNA and mRNA signatures from an hESC-based in vitro model of pancreatic differentiation reveal novel regulatory interactions. Laurent LC

rabbit anti-chromogranin (Abcam, ab15160)
Validation:: Validated in western blotting
References (of 173 listed):
• Yagishita Y et al. Constitutive Activation of Nrf2 in Mice Expands Enterogenesis in Small Intestine Through Negative Regulation of Math1. Cell Mol Gastroenterol Hepatol 11:503-524 (2021).PubMed: 32896624
• Chen Q et al. Paneth cell-derived growth factors support tumorigenesis in the small intestine. Life Sci Alliance 4:N/A (2021).PubMed: 33372038
• Jones BC et al. Paediatric gastric organoids as a tool for disease modelling and clinical translation. Pediatr Surg Int 37:317-324 (2021).PubMed: 33495862
• Quintero M et al. Cdk5rap3 is essential for intestinal Paneth cell development and maintenance. Cell Death Dis 12:131 (2021).PubMed: 33504792
• Yamazaki D et al. Role of adenomatous polyposis coli in proliferation and differentiation of colon epithelial cells in organoid culture. Sci Rep 11:3980 (2021).

rat anti- c-peptide (DSHB, GN-ID4)
Validation: “ This antibody recognizes the C-peptide (aa 33-63 of proinsulin) which separates insulin B chain (aa 1-30) from insulin A chain (aa 66-86) in the proinsulin protein (minus signal peptide sequence). Stains C-peptide in mature granules and proinsulin in immature granules of islet beta-cells. The antibody does not cross-react with rodent C-peptide/proinsulin.”

Referenes (from 15 listed):
• Tissue-specific expression of transfected human insulin genes in pluripotent clonal rat insulinoma lines induced during passage in vivo. Steiner DF. Proceedings of the National Academy of Sciences of the United States of America 85.18 (1988 Sep): 6652-6.
• Proinsulin-specific monoclonal antibodies. Immunocytochemical application as beta-cell markers and as probes for conversion. Madsen OD. Diabetes 36.10 (1987 Oct): 1203-11.
• Polyclonal origin of pancreatic islets in aggregation mouse chimaeras. Jami J. Development (Cambridge, England) 112.4 (1991 Aug): 1115-21.
• Ocular angiostrongyliasis in Semarang, Central Java. Margono SS. The American journal of tropical medicine and hygiene 26.1 (1977 Jan): 72-4.
• Efficient induction of pancreatic alpha cells from human induced pluripotent stem cells by controlling the timing for BMP antagonism and activation of retinoic acid signaling. Okochi H. PloS one 16.1 (2021): e0245204.

mouse anti-glucagon (Sigma, G2654).
Validation: “Specificity: Monoclonal Anti-Glucagon reacts with pancreatic glucagon in RIA and immunocytochemistry. The affinity constant of 6.1 x 10(8) L/M in RIA. The antibody weakly cross-reacts with gut glucagon (enteroglucagon) in an immunohistological assay. Cross-reactivity has been observed with glucagon-containing cells in fixed sections of pancreas from human, porcine, dog, rabbit, mouse, rat, guinea pig, and cat. The antibody reacts specifically against pancreatic glucagon and exhibits only very weak cross-reaction with gut glucagon (enteroglucagon). May be used for the immunohistochemical staining of Bouin’s-fixed, and formalin-fixed, paraffin-embedded pancreatic tissue sections. Binds to glucagon with an affinity constant of 6.1 × 108 M-1 in RIA. Monoclonal anti-Glucagon antibody can be used as an analytical tool for quantification of the hormone. It can also be used for immunocytochemical staining of formalin fixed and Bouin-fixed, paraffin-embedded pancreatic tissue sections. Mouse anti-Glucagon antibody reacts specifically with pancreatic glucagon. The product has also shown cross reactivity with glucagon-containing cells in fixed sections of pancreas from dog, mouse, rat, rabbit, porcine, guinea pig, cat and human and weak cross reactivity for gut glucagon (enteroglucagon).”

References (selected):
• Glucagonoma syndrome: a review and update on treatment. John A M and Schwartz R A. Journal of the European Academy of Dermatology and Venereology : JEADV, 30(12), 2016-2022 (2016)
• Islet specific Wnt activation in human type II diabetes. Lee S H, et al. Experimental Diabetes Research, 2008 (2009)
• Isolation and purification of rat islet cells by flow cytometry. Akbarzadeh A, et al. Indian Journal of Clinical Biochemistry : IJCB, 23(1), 57-57 (2008)
• The metabolic actions of glucagon revisited. Habegger K M, et al. Nature reviews. Endocrinology, 6(1), 689-689 (2010)
• The Human Glucagon Gene Is Located on Chromosome 2. Tricoli J V, et al. Diabetes, 33(2), 200-202 (1984)

donkey anti-goat Alexa Fluor 488 (Invitrogen, A-11055, 1:1,000)
Validation: “Goat IgG (H+L) Cross-Adsorbed Secondary Antibody (A-11055) in Flow
Ishikawa cells (human endometrial adenocarcinoma cell line) were cultured according to standard protocol. The culture medium was aspirated and cells rinsed with Ca and Mg free HBSS. Cells were treated with 0.25% Trypsin and incubated at 37° for 5 minutes. Cells were aspirated and pelleted at 900 x g for 5 minutes. Cells were washed twice with PBS. The cells were then fixed with 4% paraformaldehyde in PBS for 15 minutes at room temperature. The cells were then washed as stated previously. Permeabilization and blocking was performed by incubating in 5% BSA and 0.1% Triton-X in PBS for 20 minutes at room temperature. The cells were then washed as previously stated. The primary antibody for OXTR (Product # PA5-19038) was used at a 1:200 dilution in a 5% BSA, PBS solution and incubated for 120 minutes at room temperature. The cells were washed as stated previously. The secondary Alexa 488 antibody (Product # A-11055) was used at a 1:2000 dilution in 5% BSA, PBS and incubated in the dark for 45 minutes. The cells were washed and resuspended in PBS and analyzed through flow cytometry. Data courtesy of the Antibody Data Exchange Program.”
References (out of >200):
• Frontiers in neuroscience. “Induction of NTPDase1/CD39 by Reactive Microglia and Macrophages Is Associated With the Functional State During EAE.” Jakovljevic M,Lavrnja I,Bozic I,Milosevic A,Bjelobaba I,Savic D,Sévigny J,Pekovic S,Nedeljkovic N,Laketa D. 2020

• Nature communications. " DDX5 plays essential transcriptional and post-transcriptional roles in the maintenance and function of spermatogonia." Legrand JMD,Chan AL,La HM,Rossello FJ,Änkö ML,Fuller-Pace FV,Hobbs RM. 2019.

• Journal of neuroinflammation. “Mesenchymal stem cells alleviate the early brain injury of subarachnoid hemorrhage partly by suppression of Notch1-dependent neuroinflammation: involvement of Botch.” Liu W,Li R,Yin J,Guo S,Chen Y,Fan H,Li G,Li Z,Li X,Zhang X,He X,Duan C. 2019.

donkey anti-rabbit Alexa Fluor 488 (Invitrogen, A-21206, 1:1,000)

Validation: “Rabbit IgG (H+L) Highly Cross-Adsorbed Secondary Antibody (A-21206) in IHC
Immunofluorescence analysis of DA neurons using anti-tyrosine hydroxylase (TH) antibody. H9 ESCs were differentiated with PSC Dopaminergic neuron differentiation kit. H9 ESCs were specified to become midbrain floor plate (FP) progenitors which were further expanded and cryopreserved. Recovered FP progenitors were then matured for additional 14 days. Expression of TH was labeled with 1st antibody, anti-TH (Product # P21962) followed by 2nd antibody AlexaFluor488 donkey anti-rabbit (Product # A-21206, green). Nuclear DNA was stained with DAPI (blue).”

References (out of 543 references):
• Frontiers in neuroscience. “Induction of NTPDase1/CD39 by Reactive Microglia and Macrophages Is Associated With the Functional State During EAE.” Jakovljevic M,Lavrnja I,Bozic I,Milosevic A,Bjelobaba I,Savic D,Sévigny J,Pekovic S,Nedeljkovic N,Laketa D. 2020

• The Journal of clinical investigation. Lkb1 deletion in periosteal mesenchymal progenitors induces osteogenic tumors through mTORC1 activation. Han Y,Feng H,Sun J,Liang X,Wang Z,Xing W,Dai Q,Yang Y,Han A,Wei Z,Bi Q,Ji H,Kang T,Zou W. 2019

• Cell communication and signaling. “bFGF-mediated pluripotency maintenance in human induced pluripotent stem cells is associated with NRAS-MAPK signaling.” Haghighi F,Dahlmann J,Nakhaei-Rad S,Lang A,Kutschka I,Zenker M,Kensah G,Piekorz RP,Ahmadian MR. 2018

donkey anti-rat Alexa Fluor 488 (Invitrogen, A-21208, 1:1,000)
Validation: “Rat IgG (H+L) Highly Cross-Adsorbed Secondary Antibody (A-21208) in ICC/IF
Immunofluorescence analysis of Donkey anti-Rat IgG (H+L) Secondary Antibody, Alexa Fluor 488 conjugate was performed using A549 cells stained with alpha Tubulin (YL1/2) Rat Monoclonal Antibody (Product # MA1-80017). The cells were fixed with 4% paraformaldehyde for 10 minutes, permeabilized with 0.1% Triton™ X-100 for 10 minutes, blocked with 1% BSA for 1 hour and labeled with 2µg/mL Rat primary antibody for 3 hours at room temperature. Donkey anti-Rat IgG (H+L) Secondary Antibody, Alexa Fluor 488 conjugate (Product # A-21208) was used at a concentration of 1µg/mL in phosphate buffered saline containing 0.2 % BSA for 45 minutes at room temperature, for detection of alpha Tubulin in the cytoplasm (Panel a: green). Nuclei (Panel b: blue) were stained with DAPI in SlowFade® Gold Antifade Mountant (Product # S36938). F-actin was stained with Rhodamine Phalloidin (Product # R415, 1:300) (Panel c: red). Panel d represents the composite image. No nonspecific staining was observed with the secondary antibody alone (panel f), or with an isotype control (panel e). The images were captured at 60X magnification.”

References (of 142 references):
• Nature communications. IFN-β is a macrophage-derived effector cytokine facilitating the resolution of bacterial inflammation. Kumaran Satyanarayanan S,El Kebir D,Soboh S,Butenko S,Sekheri M,Saadi J,Peled N,Assi S,Othman A,Schif-Zuck S,Feuermann Y,Barkan D,Sher.N,Filep JG,Ariel A. 2019.

• Nature communications. The PDGF-BB-SOX7 axis-modulated IL-33 in pericytes and stromal cells promotes metastasis through tumour-associated macrophages. Yang Y,Andersson P,Hosaka K,Zhang Y,Cao R,Iwamoto H,Yang X,Nakamura M,Wang J,Zhuang R,Morikawa H,Xue Y,Braun H,Beyaert R,Samani N,Nakae S,Hams E,Dissing S,Fallon PG,Langer R,Cao Y. 2016.

donkey-anti-mouse-Alexa Fluor 647 (A-31571, 1:1,000)
Validation: “Mouse IgG (H+L) Highly Cross-Adsorbed Secondary Antibody (A-31571) in ICC/IF. Immunofluorescence analysis of Donkey anti-Mouse IgG (H+L) Highly Cross-Adsorbed Secondary Antibody, Alexa Fluor® 647 conjugate was performed using HeLa cells stained with alpha Tubulin (236-10501) Mouse Monoclonal Antibody (Product # A11126). The cells were fixed with 4% paraformaldehyde for 10 minutes, permeabilized with 0.1% Triton™ X-100 for 10 minutes, blocked with 1% BSA for 1 hour and labeled with 2 µg/mL primary antibody for 3 hours at room temperature. Donkey anti-Mouse IgG (H+L) Highly Cross-Adsorbed Secondary Antibody, Alexa Fluor® 647 (Product # A-31571) was used at a concentration of 2 µg/mL in phosphate buffered saline containing 0.2% BSA for 45 minutes at room temperature, for detection of alpha Tubulin in the cytoplasm (Panel a: red). Nuclei (Panel b: blue) were stained with DAPI in SlowFade® Gold Antifade Mountant (Product # S36938). F-actin was stained with Alexa Fluor® 488 Phalloidin (Product # A12379), 1:300) (Panel c: green). Panel d represents the composite image. No nonspecific staining was observed with the secondary antibody alone (panel f), or with an isotype control (panel e). The images were captured at 60X magnification.”

References (Out of 142):

• Journal of neurochemistry. The neuroprotective effect of latanoprost acts via klotho-mediated suppression of calpain activation after optic nerve transection. Yamamoto K,Sato K,Yukita M,Yasuda M,Omodaka K,Ryu M,Fujita K,Nishiguchi KM,Machida S,Nakazawa T. 2017

• eLife. Kindlin-2 cooperates with talin to activate integrins and induces cell spreading by directly binding paxillin. Theodosiou M,Widmaier M,Böttcher RT,Rognoni E,Veelders M,Bharadwaj M,Lambacher A,Austen K,Müller DJ,Zent R,Fässler R. 2016.

• Nature cell biology. A human genome-wide screen for regulators of clathrin-coated vesicle formation reveals an unexpected role for the V-ATPase. Kozik P,Hodson NA,Sahlender DA,Simecek N,Soromani C,Wu J,Collinson LM,Robinson MS. 2013.

Eukaryotic cell lines

Policy information about cell lines

Cell line source(s)	HUES8 embryonic cell line from Douglas Melton/Harvard Stem Cell Institute at Harvard University
Authentication	This cell line is a NIH registered human embryonic cell line (NIHhESC-09-0021) that comes from the original source (Douglas Melton) and is available from the Harvard Stem Cell Institute (HSCI). Stem cell cultures were genotyped by STR profile and karyotyped regularly to ensure validity of cell lines used.
Mycoplasma contamination	All cell lines were regularly tested for mycoplasma contamination and tested negative.
Commonly misidentified lines (See ICLAC register)	None

Animals and other organisms

Policy information about studies involving animals; ARRIVE guidelines recommended for reporting animal research

Laboratory animals	Islet donors: C57BL/6 female retired breeders (age not specified/determined as these are random aged retired breeders);<br>Islet recipients: C57BL/6 mice (6-8 weeks old male);<br>Human islet recipients: NOD-scid-Il2rgc-/- (NSG) (Jackson Laboratory, Bar Harbor, ME) mice (age 6-12 weeks, male)<br>Porcine islet recipients: NSG mice (age 6-12 weeks, mixture of male and female)
Wild animals	no wild animals were used in this study.
Field-collected samples	no field-collected samples were used in this study
Ethics oversight	Institutional IACUC committees from the University of Minnesota (protocol #1905-37028A) and the Mayo Clinic (protocol #A00003973) approved the animal studies.

Note that full information on the approval of the study protocol must also be provided in the manuscript.

Flow Cytometry

Plots

Confirm that:

☒ The axis labels state the marker and fluorochrome used (e.g. CD4-FITC).

☒ The axis scales are clearly visible. Include numbers along axes only for bottom left plot of group (a 'group' is an analysis of identical markers).

☒ All plots are contour plots with outliers or pseudocolor plots.

☒ A numerical value for number of cells or percentage (with statistics) is provided.

Methodology

Sample preparation	SC-beta cell clusters were dissociated into single cells, permeabilized, and stained wiht primary and secondary antibodies as fully described in the materials and methods.
Instrument	ThermoFisher Attune flow cytometer
Software	NXT v. 3.1 (ThermoFisher) and FlowJo v.10 (FlowJo, Ashland, OR)
Cell population abundance	Cell sorting was not used in this study
Gating strategy	All captured events are first gated by forward scatter area, side scatter area. Doublet discrimination is performed to define all singlets by gating along the diagonal of forward scatter height, forward scatter width. Final flow cytometry plots for SC-beta characterization are shown as contour plots, with outliers shown. For beta cell specific viability, four-quadrant dot plots are presented with live and dead populations defined from the live/dead marker v. forward scatter area.

☒ Tick this box to confirm that a figure exemplifying the gating strategy is provided in the Supplementary Information.
